# A Detuning-Resilient Wideband Microstrip Antenna for Wearable Biomedical Telemetry Through Progressive Interdigital Slot Integration and Patch Defect Engineering

**DOI:** 10.3390/s26196117

**Published:** 2026-09-26

**Authors:** Prince O. Siaw, Ebenezer Adjei, Ahmad Aldelemy, John G. Buckley, Raed Abd-Alhameed

**Affiliations:** 1 Institute of Digital and Sustainable Futures, University of Bradford, Bradford BD7 1DP, UK; p.o.siaw@bradford.ac.uk (P.O.S.); e.adjei@bradford.ac.uk (E.A.); a.a.aldelemy@bradford.ac.uk (A.A.); 2 Management, Sciences and Engineering, University of Bradford, Bradford BD7 1DP, UK; j.buckley@bradford.ac.uk; 3 Department of Information and Communication Engineering, Alfarqadein University, Basrah 61004, Iraq

**Keywords:** bandwidth enhancement, circular microstrip patch antenna, compact antenna, defected patch structure, equivalent circuit model, interdigital slots, sub-6 GHz, wideband antenna, detuning resilience, specific absorption rate

## Abstract

This paper presents the systematic design, equivalent circuit analysis, and experimental validation of a compact wideband circular microstrip patch antenna for use in biomedical telemetry. The antenna, loaded with interdigital capacitive slots and a defected patch structure (DPS), was fabricated on a Rogers RO3006 substrate (relative permittivity 6.5, loss tangent 0.002, thickness 0.8 mm) with overall dimensions of 25 × 36.96 mm^2^. A stepwise design evolution through five variants (ANT1–ANT5) illustrates the progressive bandwidth enhancement mechanisms. The final design (ANT5) achieved a simulated −10 dB impedance bandwidth of 1.50 GHz (2.51–4.01 GHz), corresponding to a fractional bandwidth of 46%. The fabricated prototype exhibited a measured −10 dB bandwidth of 1.42 GHz (2.54–3.96 GHz), representing a fractional bandwidth of 43.7%. An equivalent lumped-element circuit model was derived and validated against the full-wave simulation results. The antenna exhibited a simulated peak free-space gain of 3.2 dBi at 3.8 GHz, with free-space radiation efficiency exceeding 90%. Under multi-layer tissue phantom loading, the antenna maintained a robust 910 MHz operational bandwidth with a safe maximum specific absorption rate (SAR) of 0.724 W/kg and an on-body efficiency of 8.4%. The compact size and wide fractional bandwidth provide enhanced resilience against environmental detuning, making this design particularly suitable for robust wearable biomedical telemetry.

## 1. Introduction

Microstrip patch antennas (MPAs) continue to be preferred radiating elements for modern wireless communication platforms owing to their low profile, lightweight construction, ease of integration with monolithic microwave integrated circuits (MMICs), and conformability to both planar and non-planar surfaces [1,2,3]. With the rapid expansion of fifth generation (5G) New Radio (NR) sub-6 GHz bands, Wi-Fi 6/6E, the Internet of Things (IoT), and particularly, wireless body area networks (WBANs) for continuous healthcare monitoring, there is increasing demand in biomedical telemetry for compact antennas that can simultaneously cover multiple frequency bands or operate over a wide continuous bandwidth [4,5,6]. This demand is particularly critical for emerging wireless body area networks (WBANs) and wearable biomedical sensing, where continuous healthcare monitoring requires antennas that maintain robust performance when deployed on the human body [7,8]. However, conventional MPAs inherently suffer from narrow impedance bandwidth, typically ranging from 1% to 5%, which significantly limits their applicability in wideband communication systems [9,10]. This limitation becomes particularly pronounced when high-permittivity substrates are employed for antenna miniaturisation, as the increased effective electrical thickness and tighter electromagnetic coupling to the ground plane further constrain achievable bandwidth [11,12].

Over the past decade, numerous techniques have been investigated to enhance the impedance bandwidth of MPAs. These include thick low-permittivity substrates [13], stacked or multilayer patch configurations [14,15], aperture-coupled feeding mechanisms [16], U-slot loading [17,18], L-probe and Γ-probe feeding [19], E-shaped and H-shaped patches [20,21], defected ground structures (DGS) [22,23,24,25], metasurface-based approaches [26,27], substrate-integrated waveguide (SIW) techniques [28], and parasitic element coupling [29]. While effective, many of these techniques increase the antenna’s vertical profile, design complexity, fabrication cost, or overall footprint, which may be undesirable for compact, low-cost, and system-integrated applications.

Slot loading directly on the radiating patch is a particularly attractive alternative because it enables bandwidth enhancement and effective size reduction without increasing the antenna’s vertical profile [30,31,32,33]. Among various slot configurations, interdigital (or comb-line) slots have garnered significant research attention due to their ability to introduce distributed capacitive loading, which effectively lengthens the current path, lowers the resonant frequency, and can create additional closely spaced resonances that merge with the fundamental mode to substantially broaden the impedance bandwidth [34,35,36]. The interdigital slot geometry provides a high degree of design flexibility, as the number of fingers, finger length, finger width, inter-finger gap, and relative finger offset can all be independently optimised to tailor the antenna’s impedance and radiation characteristics.

The concept of a defected patch structure (DPS), the deliberate removal of a portion of the conducting patch is analogous to the well-established DGS technique but applied to the radiating element itself [37]. DPS modifies the surface current distribution on the patch, introduces additional inductance and capacitance, and can excite higher-order or perturbed modes that contribute to bandwidth widening. Recent studies have demonstrated the effectiveness of DPS in achieving multiband and wideband behaviour in compact patch antennas [38,39]. When combined with interdigital slot loading, DPS offers a powerful synergistic mechanism for achieving wideband performance in a compact form factor, as the distributed capacitive loading from the interdigital slots and the localised inductive perturbation from the DPS create a broadband impedance matching condition directly within the radiating structure.

In this paper, a compact wideband circular MPA employing bilateral interdigital capacitive slots and a central rectangular DPS is proposed specifically for robust wearable biomedical telemetry within wireless body area networks (WBANs). The antenna is designed on a Rogers RO3006 substrate (εᵣ = 6.5, h = 0.8 mm), a high-permittivity material that provides inherent miniaturisation, while the substrate thickness partially compensates for the bandwidth limitation imposed by high permittivity. A systematic five-step design evolution (ANT1 through ANT5) is presented, wherein each iteration introduces an additional structural modification to the patch, and the resulting impact on impedance bandwidth is rigorously analysed through full-wave electromagnetic simulation. A parametric study was conducted to demonstrate the sensitivity of key design parameters and justify the final optimised dimensions. An equivalent lumped-element circuit model was developed and validated against full-wave simulation results. The final antenna prototype was fabricated and experimentally characterised through reflection coefficient measurements, with results demonstrating a −10 dB impedance bandwidth spanning approximately 2.54–3.96 GHz (~43.7% fractional bandwidth).

The principal contributions of this work are summarised as follows:

1. A systematic, step-by-step design methodology that clearly delineates the individual and cumulative effects of interdigital slots and DPS on the bandwidth of a circular MPA on a high-permittivity substrate;
2. A comprehensive parametric study demonstrating the sensitivity of bandwidth to key geometric parameters, providing design guidelines for adaptation to other frequency bands;
3. An equivalent lumped-element circuit model validated against full-wave simulation, providing circuit-level understanding of the electromagnetic phenomena introduced by each structural modification;
4. Comprehensive full-wave simulation analysis including reflection coefficient evolution, surface current distribution, radiation patterns, gain, and efficiency;
5. Experimental validation through prototype fabrication and S-parameter measurement, demonstrating good agreement with simulation;
6. Achievement of wideband performance (>43% measured fractional bandwidth) on a high-permittivity (εᵣ = 6.5), moderately thin (0.8 mm ≈ 0.0075λ_0_ at 3.25 GHz) substrate with a compact footprint of 25 × 36.96 mm^2^ (0.27λ_0_ × 0.40λ_0_);
7. Evaluation of the antenna’s robustness and safety for wearable applications, demonstrating excellent detuning immunity and specific absorption rate (SAR) compliance on a multi-layer human tissue phantom.

The remainder of this paper is organised as follows. Section 2 describes the antenna geometry and design parameters. Section 3 presents the stepwise design evolution. Section 4 develops the parametric study. Section 5 presents the equivalent circuit model. Section 6 discusses simulation results in free space. Section 7 presents the biomedical telemetry validation and safety evaluation. Section 8 provides experimental validations of the fabricated prototype. Section 9 provides a performance comparison with state-of-the-art designs. Section 10 concludes the paper.

## 2. Antenna Geometry and Design Parameters

### 2.1. Substrate Selection and Justification

The antenna has been designed on a Rogers RO3006 (Rogers Corporation, Everghem, Belgium) substrate with the following material specifications: relative permittivity εᵣ = 6.5 ± 0.15 (manufacturer tolerance), loss tangent tan δ = 0.002 at 10 GHz, relative permeability μᵣ = 1, substrate thickness h = 0.8 mm, and thermal conductivity κ = 0.79 W/(K·m). The copper cladding is 1 oz (35 μm) annealed copper with electrical conductivity σ = 5.8 × 10^7^ S/m.

The selection of RO3006 substrate was motivated by several considerations:

(1) *Miniaturisation:* The high relative permittivity (εᵣ = 6.5) reduces the guided wavelength according to λg = λ_0_/√εeff, enabling a significantly smaller patch radius for a given operating frequency compared to standard FR-4 (εᵣ ≈ 4.4) or low-permittivity substrates such as Rogers RT/duroid 5880 (εᵣ = 2.2);
(2) *Low loss:* The very low loss tangent (tan δ = 0.002) ensures that dielectric losses remain minimal, preserving radiation efficiency despite the high permittivity;
(3) *Substrate thickness:* The 0.8 mm thickness represents a practical compromise thin enough to maintain a low-profile structure (h/λ_0_ ≈ 0.0087 at 3.25 GHz) while sufficiently thick to support the additional reactive loading introduced by interdigital slots and DPS without an excessively high Q-factor;
(4) *Mechanical stability:* RO3006 provides excellent dimensional stability across temperature variations, important for maintaining consistent antenna performance in practical deployment scenarios;
(5) *Tissue Coupling*: The selection of RO3006 ($\epsilon_{r}=6.5$) not only aids in miniaturisation but also provides a better impedance bridge between the antenna and biological media compared to low-permittivity substrates. This reduces signal reflection at the tissue-antenna interface, which is advantageous for maintaining reliable signal coupling in on-body telemetry applications.

### 2.2. Antenna Configuration

The proposed antenna, designated ANT5 in the design evolution sequence, consists of a circular radiating patch of diameter D = 17.16 mm (radius a = 8.58 mm) printed on the top copper layer of the substrate. The patch is fed by a microstrip transmission line of width Wf = 0.12 mm and length Lf = 15.97 mm. The feed line connects to the patch through a wider stub/taper section of width Ws = 2 mm, providing an impedance transition between the narrow high-impedance feed line and the lower-impedance patch edge. The geometry and detailed dimensions of the proposed antenna (ANT5) are illustrated in Figure 1, with all design parameters summarised in Table 1.

The ground plane is a coplanar partial (truncated) ground located on the top surface, flanking the feed line, with dimensions of 5.69 mm × 6.65 mm, positioned beneath the lower portion of the feed line. The partial ground plane serves multiple purposes:

(1) It reduces the quality factor (Q) of the antenna by decreasing stored electromagnetic energy relative to radiated energy;
(2) It facilitates wideband impedance matching by allowing the feed line to act partially as a transmission line and partially as a radiating element;
(3) It creates a smooth transition between the guided-wave region and the radiating region.

The overall substrate dimensions are W_sub_ = 25 mm × L_sub_ = 36.96 mm, corresponding to approximately 0.27λ_0_ × 0.40λ_0_ at the centre frequency of 3.25 GHz.

### 2.3. Interdigital Slot Configuration

The circular patch is loaded with bilateral interdigital slots forming the primary bandwidth enhancement mechanism. These consist of two sets of parallel horizontal slot fingers extending inward from opposite edges of the circular patch. The slot fingers from each side are interleaved, forming an interdigitated capacitive structure. The key slot parameters are shown in Table 1.

A central rectangular slot constituting the DPS is etched in the upper portion of the circular patch, coinciding with the feed point transition. This deliberate defect serves the following purposes:

(1) It further perturbs the surface current distribution by forcing currents to detour around defect boundaries;
(2) It introduces additional series inductance in the primary current path;
(3) It creates a secondary resonant mode that merges with the fundamental to produce a wideband response;
(4) It provides an additional degree of freedom for fine-tuning input impedance. The complete set of design parameters for the final antenna (ANT5) is summarised in Table 1.

## 3. Stepwise Design Evolution

The final antenna design (ANT5) was developed through a systematic five-step evolution in Figure 2. Each step introduces a specific structural modification, enabling clear identification of each feature’s contribution to wideband performance. The simulated reflection coefficient (|S_11_|) responses of all five variants are compared in Figure 3.

### 3.1. ANT 1: Reference Circular Patch with Single Slot

ANT1 serves as the baseline reference design, consisting of a conventional circular microstrip patch (D = 17.16 mm) with a single rectangular slot near the feed point.

The fundamental resonant frequency of the unperturbed circular MPA can be estimated using the cavity model [1]:

(1) $$f_{mn}=\frac{X_{mn}^{\prime }\cdot c}{2\pi a_{e}\sqrt{\varepsilon_{r}}}$$

where *X*′*_mn_* is the *m-th* zero of the derivative of the Bessel function of the first kind of order *n* (*X*′_11_ = 1.8412 for the dominant TM_11_ mode), *c* = 3 × 10^8^ m/s is the speed of light in free space, and $a\varepsilon_{r}$ is the effective radius accounting for fringing fields at the patch periphery.

(2) $$a_{e}=a\sqrt{1+\frac{2h}{\pi a\varepsilon_{r}}\left[ln\left(\frac{\pi a}{2h}\right)+1.7726\right]}$$

Substituting *a* = 8.58 mm, *h* = 0.8 mm, and *εᵣ* = 6.5 yields *a_e_* ≈ 8.76 mm and *f*_11_ ≈ 3.94 GHz. However, the single slot perturbation, feed structure, and partial ground plane collectively lower the effective resonant frequency to approximately 2.8 GHz.

The simulated |S_11_| of ANT1 shows a resonance near 2.8 GHz with a −10 dB bandwidth of approximately 280 MHz (fractional bandwidth ~10%).

### 3.2. ANT 2: Addition of Left-Side Interdigital Slots

In ANT2, horizontal interdigital slot fingers are introduced on the left half of the circular patch, creating a unilateral combo-line structure. This modification produces the following:

- Current path elongation: Surface currents meander around slot fingers, increasing electrical length;
- Reactive loading: Additional distributed inductance and capacitance modify input impedance;
- Bandwidth improvement: −10 dB bandwidth.

### 3.3. ANT 3: Bilateral Interdigital Slot Configuration

ANT3 extends interdigital slot loading to both sides, creating a symmetric bilateral configuration with a 0.5 mm gap between opposing fingertips. This produces the following:

- Enhanced distributed capacitance: Coupling between opposing fingertips introduces substantial inter-digital capacitance;
- Symmetric current distribution improves radiation pattern symmetry;
- Further bandwidth broadening: The −10 dB bandwidth increases to approximately 780 MHz (~27% fractional bandwidth).

### 3.4. ANT 4: Introduction of Central Defected Patch Structure

In ANT4, a central rectangular defect (DPS) is introduced near the feed transition. Effects include the following:

- Additional series inductance: Currents flow around defect boundaries;
- Secondary resonance excitation creates a resonant mode slightly offset from the fundamental;
- Bandwidth enhancement: The −10 dB bandwidth increases to approximately 1.08 GHz (~37% fractional bandwidth).

### 3.5. ANT 5: Optimised Final Design

ANT5 represents the final optimised design, achieved through parametric optimisation in CST Microwave Studio. The optimisation targets were as follows:

1. Minimize |S_11_| below −10 dB over the widest possible bandwidth;
2. Maintain broadside radiation with peak gain > 2 dBi;
3. Keep overall dimensions within 25 × 36.96 mm^2^.

The simulated |S_11_| of ANT5 exhibited the widest −10 dB bandwidth of 1.50 GHz (2.51–4.01 GHz), corresponding to 46% fractional bandwidth. Table 2 summarises the progressive bandwidth enhancement.

## 4. Parametric Study

A comprehensive parametric study was conducted to investigate the sensitivity of antenna performance to key geometric parameters. This analysis justifies the final optimised dimensions and provides design guidelines for adaptation to other applications.

### 4.1. Effect of Slot Finger Length ($l_{f}$)

The finger length controls both the inductive path length and the capacitive coupling area. Figure 4a shows |S_11_| for finger lengths from 3.5 mm to 5.5 mm.

Observations:

- $l_{f}$ = 3.5 mm: Insufficient loading; bandwidth limited to 1.1 GHz; poor matching at lower frequencies.
- $l_{f}$ = 4.68 mm (optimal): Maximum bandwidth with smooth impedance transition across the band.
- $l_{f}$ = 5.5 mm: Excessive loading; resonance shifts significantly lower; bandwidth reduces due to increased Q-factor from stronger reactive loading.

### 4.2. Effect of Inter-Finger Gap (g)

The inter-finger gap between opposing slot fingers critically controls the inter-digital capacitance. Figure 4b shows the simulated |S_11_| for gap values ranging from 0.3 mm to 0.9 mm.

Observations:

- *g* = 0.3 mm: Excessive capacitive loading shifts resonance downward to 2.4 GHz; bandwidth narrows to 1.2 GHz due to over-coupling.
- *g* = 0.5 mm (optimal): Balanced capacitive loading provides maximum bandwidth of 1.50 GHz with good matching (|S_11_| < −25 dB).
- *g* = 0.7 mm: Reduced capacitance shifts resonance upward; bandwidth decreases to 1.35 GHz.
- *g* = 0.9 mm: Insufficient coupling; bandwidth reduces to 1.15 GHz; secondary resonance becomes distinct rather than merged.
- The inter-digital capacitance varies approximately as Cid ∝ 1/g for small gaps, explaining the strong sensitivity observed.

### 4.3. Effect of DPS Dimensions

The DPS geometry affects the secondary resonance frequency and the inductive loading magnitude. Figure 4c shows |S_11_| variation with DPS length.

Observations:

- Increasing DPS length increases the series inductance, lowering the secondary resonance frequency.
- Optimal DPS dimensions position the secondary resonance approximately 400 MHz above the primary resonance, enabling effective merging.
- Excessively large DPS creates a distinct dual-band response rather than a merged wideband response.

### 4.4. Effect of Partial Ground Length ($L_{g}$)

The partial ground length affects the transition between guided-wave and radiating-wave behaviour. Figure 4d shows the sensitivity analysis.

Observations:

- $L_{g}$ < 5 mm: Insufficient ground; poor low-frequency matching; radiation pattern degradation.
- $L_{g}$ = 5.69 mm (optimal): Balanced transition providing wideband matching.
- $L_{g}$ > 7 mm: Extended ground increases the Q-factor, reducing bandwidth.

## 5. Equivalent Circuit Model

### 5.1. Motivation and Approach

To develop physical insight into the wideband behaviour and provide a computationally efficient design tool, an equivalent lumped-element circuit model was derived. This model is based on the transmission line representation of the microstrip patch antenna, augmented with lumped elements representing the interdigital slots and DPS.

### 5.2. Basic Circular Patch Circuit (ANT1 Basis)

The basic circular patch resonator operating in the dominant TM_11_ mode can be represented as a parallel RLC circuit [1,40]:

(3) $$Z_{patch}=\frac{1}{\frac{1}{R_{p}}+j\omega C_{p}+\frac{1}{j\omega L_{p}}}$$

where Patch capacitance ($C_{p}$) represents the electric energy stored in the dielectric between the patch and the ground plane:

(4) $$C_{p}=\frac{\varepsilon_{0}\varepsilon_{r}\pi a_{e}^{2}}{2h}$$

For the RO3006 substrate with h = 0.8 mm:

$$C_{p}=\frac{8.854\times 10^{-12}\times 6.5\times \pi \times (8.76\times 10^{-3}{)}^{2}}{2\times 0.8\times 10^{-3}}$$

(5) $$C_{p}=\frac{8.854\times 10^{-12}\times 6.5\times 2.41\times 10^{-4}}{1.6\times 10^{-3}}\approx 8.67\;\mathrm{pF}\;$$

Patch inductance ($L_{p}$) is determined from the resonance condition:

(6) $$L_{p}=\frac{1}{\left(2\pi f_{0}{)}^{2}C_{p}\right.}$$

For f_0_ = 2.8 GHz:

(7) $$L_{p}=\frac{1}{\left(2\pi \times 2.8\times 10^{9}{)}^{2}\times 8.67\times 10^{-12}\right.}\approx 0.373\;\mathrm{nH}\;$$

Radiation resistance ($R_{p}$) represents the radiated power and determines the unloaded Q-factor.

The unloaded Q-factor for a circular patch on a substrate with εᵣ = 6.5 and h = 0.8 mm can be estimated as Q_u_ ≈ 30–50. Taking Q_u_ ≈ 40:

(8) $$R_{p}=\frac{40}{2\pi \times 2.8\times 10^{9}\times 8.67\times 10^{-12}}\approx 262\;\Omega$$

### 5.3. Interdigital Slot Circuit Model

The bilateral interdigital slots are modelled as a series LC network.

Interdigital capacitance (*C_id_*) using conformal mapping is expressed as follows [41,42]:

(9) $$C_{id}=(\varepsilon_{0}\varepsilon_{r}+1)\frac{K\left(k\right)}{K\left(k^{\prime }\right)}l_{f}\left(N-1\right)$$

where $K\left(k\right)$ is the complete elliptic integral of the first kind, and N=15 represents the precise number of interdigital fingers per side. While this analytical formulation establishes the physical foundation for the interdigital capacitance, high-permittivity substrates ($\epsilon_{r}=6.5$) introduce complex fringing fields and parasitic cross-coupling not fully captured by ideal equivalent equations. Consequently, the initial analytical values were refined through bounded curve-fitting to match the full-wave electromagnetic response, ensuring the lumped-element model accurately represents the physical prototype’s true wideband mode-merging mechanism.

Interdigital inductance (*L_id_*) accounting for current path elongation is expressed as follows:

(10) $$L_{id}=\mu_{0}\frac{l_{f}\cdot N}{w_{finger}}\cdot h_{eff}\;$$

Extracted values are *C_id_* ≈ 0.42 pF, *L_id_* ≈ 1.85 nH.

### 5.4. Defected Patch Structure Circuit Model

The DPS is modelled as a series combination of inductance ($L_{dps}$) and resistance ($R_{dps}$), with a parallel parasitic capacitance ($C_{dps}$):

(11) $$L_{dps}=\mu_{0}\frac{l_{dps}}{w_{dps}}\cdot h$$

(12) $$\;C_{dps}=\varepsilon_{0}\varepsilon_{r}\frac{l_{dps}\cdot w_{dps}}{h}$$

The DPS introduces a resonance at:

(13) $$f_{dps}=\frac{1}{2\pi \sqrt{L_{dps}C_{dps}}}$$

### 5.5. Complete Equivalent Circuit

The complete equivalent circuit is shown in Figure 5a, and the extracted lumped-element parameter values are summarized in Table 3. The circuit model S_11_ response is compared with full-wave simulation in Figure 5b.

The circuit model demonstrates agreement within ±1.5 dB with full-wave simulation across the 2–5 GHz band, validating the physical interpretation. To quantitatively assess this agreement, the root mean square (RMS) error between the simulated and circuit-modelled $S_{11}$ response was calculated to be ~0.42 dB, validating the physical interpretation of the antenna’s wideband merging mechanism. A discrete comparison at key operating frequencies is provided in Table 4.

The loaded Q-factor is:

(14) $${\;Q}_{loaded}=\frac{f_{0}}{\Delta f_{-10dB}}=\frac{2.79}{1.50}\approx 1.86$$

This represents a reduction from Q ≈ 40 (unloaded basic patch) to Q ≈ 1.86, a factor of approximately 21.5× improvement in bandwidth, as illustrated in Figure 6.

## 6. Simulation Results and Analysis

All full-wave electromagnetic simulations were performed using CST Microwave Studio 2024, employing the time-domain solver with adaptive hexahedral meshing. Open boundary conditions with *λ*/4 additional space were applied. The frequency range was 1–5.5 GHz with adaptive mesh refinement (convergence criterion ΔS < 0.01).

### 6.1. Motivation and Reflection Coefficient (S_11_) in Free Space

The simulated reflection coefficient of the final design (ANT5) is presented in Figure 7. The key performance metrics are as follows:

- Resonant frequency: 2.79 GHz
- Return loss at resonance: 29.4 dB
−10 dB bandwidth: 2.51–4.01 GHz (1.50 GHz)
- Fractional bandwidth: 46% (referenced to 3.26 GHz centre frequency)

### 6.2. Input Impedance Analysis

The simulated input impedance on a Smith chart (Figure 8) shows a compact loop near the centre across the 2.5–4.0 GHz band. The real part remains between 35–75 Ω, while the imaginary part oscillates between −25 Ω and +25 Ω, maintaining VSWR < 2:1.

The compact impedance loop near the centre of the Smith chart indicates a merged resonance. This characteristic is crucial for antennas, as it provides a ‘matching buffer’ that prevents the antenna from failing if environmental conditions shift the operating frequency slightly.

The real part of the impedance varies between 35–75 Ω, while the imaginary part oscillates between −25 Ω and +25 Ω. The entire impedance trajectory remains within the VSWR = 2:1 circle (green dashed), confirming wideband matching performance across the operating band.

### 6.3. Surface Current Distribution

To visualize the wideband mode-merging mechanism, the simulated surface current distributions were analyzed at the lower and upper bounds of the primary operating band (Figure 9).

At 2.8 GHz (Figure 9a), representing the fundamental mode, the highest current density is concentrated along the narrow microstrip feed and meanders through the bilateral interdigital slots. The anti-parallel current orientations across the inter-finger gaps confirm strong distributed capacitive coupling.

At 3.8 GHz (Figure 9b), representing the secondary mode, the current distribution shifts to show enhanced concentration along the edges of the central defected patch structure (DPS). This confirms that the localised inductive loading of the DPS actively facilitates the higher-order resonance. Together, these distributions substantiate the synergistic capacitive-inductive coupling that merges the dual modes into a unified wideband response.

### 6.4. Radiation Patterns

The simulated 3D far-field radiation patterns at 2.8 GHz, 3.2 GHz, and 3.8 GHz are presented in Figure 10, illustrating the antenna’s spatial radiation characteristics.

At 2.8 GHz (lower band):

- Broadside pattern with peak realized gain: 2.48 dBi
- Radiation efficiency: 94.2%
- HPBW: ~90° (E-plane), ~100° (H-plane)

At 3.2 GHz (mid-band):

- Peak realized gain: 2.87 dBi
- Radiation efficiency: 93.5%
- Total efficiency: 91.2%

At 3.8 GHz (upper band):

- Peak realized gain: 3.21 dBi
- Radiation efficiency: 91.8%
- HPBW: ~75° (E-plane), ~85° (H-plane)

The radiation patterns maintain consistent broadside characteristics across the operating band, as follows:

- Stable main beam direction (boresight)
- HPBW variation < 15° across the band
- Cross-polarization > 15 dB below co-polarization at boresight
- Front-to-back ratio > 8 dB

### 6.5. Gain and Efficiency

Figure 11 shows simulated gain and efficiency across frequency. The following values apply:

- Realised gain varies from 2.3 dBi at 2.5 GHz to 3.2 dBi at 4.0 GHz;
- Average in-band gain: 2.75 dBi;
- Radiation efficiency: >90% across entire −10 dB bandwidth;
- Total efficiency: >85% across 2.6–3.8 GHz range.

It should be noted that the average in-band gain of 2.75 dBi reflects the fundamental physical limitations of employing a high-permittivity ($\epsilon_{r}=6.5$) substrate. While low-permittivity substrates yield higher radiation aperture efficiencies and gain, the selected RO3006 substrate enforces a necessary design trade-off, prioritising extreme miniaturisation and tissue-impedance matching over absolute gain.

### 6.6. Time-Domain Characteristics

For wearable telemetry nodes employing pulse-based communication, maintaining signal integrity in the time domain is critical to prevent inter-symbol interference and pulse distortion. To evaluate the proposed design’s suitability for such applications, the transmission phase response and group delay ($\tau_{g}=-\frac{d\phi }{d\omega }$) were analyzed. A face-to-face two-antenna system was simulated to extract the forward transmission coefficient ($S_{21}$).

The simulated $S_{21}$ phase response demonstrates a highly linear profile across the entire 2.51–4.01 GHz operating band. Because group delay is the negative derivative of the phase with respect to angular frequency, this phase linearity translates directly into a stable group delay response. As illustrated in Figure 12, the group delay fluctuates gently between 0.76 ns and 1.0 ns, resulting in a maximum group delay variation ($\Delta \tau_{g}$) of strictly less than 0.25 ns throughout the −10 dB bandwidth. This minimal variation confirms that the antenna introduces negligible phase distortion and pulse spreading, verifying its excellent capability for reliable, high-data-rate pulse transmission within dynamic WBAN environments.

## 7. Biomedical Telemetry Validation

### 7.1. Tissue Phantom Loading Analysis

To validate the antenna’s suitability as a robust biomedical antenna for wearable healthcare applications [43], its performance was evaluated near human tissue. As illustrated in Figure 13, a three-layer rectangular tissue phantom consisting of skin (2 mm), fat (5 mm), and muscle (20 mm) was modelled in CST Microwave Studio with dielectric properties as described in Table 5. The antenna was placed at a 2 mm offset from the skin layer to simulate a wearable enclosure and typical clothing gap.

To simulate a standard wearable deployment on the human torso or limb, the phantom layers were assigned thicknesses of 2 mm (skin), 5 mm (fat), and 20 mm (muscle). These dimensions are consistent with established anatomical averages commonly utilized in evaluating sub-6 GHz wireless body area network (WBAN) devices.

While the proximity to the high-permittivity biological media introduced a slight frequency shift due to capacitive loading, the antenna’s inherent wide fractional bandwidth provided an excellent matching buffer. The loads remained effectively matched across the primary telemetry bands, demonstrating superior immunity to detuning compared to conventional narrow-band patches. This robust performance is verified by the loaded reflection coefficient (Figure 14), which maintained a 910 MHz operational bandwidth despite the tissue-induced frequency shift. Additionally, the simulated 3D far-field radiation pattern (Figure 15) demonstrates that while the highly lossy human tissue inherently absorbs significant electromagnetic energy, the antenna successfully maintains a functional on-body radiation efficiency of 8.4%, ensuring reliable short-range communication for wearable telemetry nodes.

### 7.2. Specific Absorption Rate (SAR) Evaluation

For wearable diagnostic antenna, verifying electromagnetic safety is critical. Because the human body acts as a highly dispersive medium, the specific absorption rate (SAR) was evaluated to ensure compliance with strict health standards. The SAR averaged over a 1 g tissue mass at the loaded resonant frequency of 3.38 GHz using the setup described in Figure 13.

With the input power normalised to 10 mW a standard transmission power for Bluetooth low energy (BLE) and medical telemetry nodes, the maximum simulated SAR is 0.724 W/kg (Figure 16). This value is well below the strict FCC safety limit of 1.6 W/kg, confirming the antenna’s viability for continuous, non-invasive on-body monitoring.

### 7.3. Detuning Resilience and Environmental Robustness

To further evaluate the antenna as a reliable biomedical antenna, a sensitivity analysis was performed regarding environmental variations common in wearable applications. The antenna’s performance is primarily dependent on the “matching buffer” provided by its 43.7% measured fractional bandwidth. In practical wearable scenarios, the distance between the antenna and the skin (air gap) can fluctuate due to body movement or varying thickness of clothing.

While a conventional narrow band microstrip patch (ANT1, 10% FBW) would experience total impedance mismatch and signal loss upon such shifts, the proposed design (ANT5) maintains operational stability. As observed in the tissue-loading simulations, the dominant resonance experienced an upward shift to 3.38 GHz. While proximity to high-permittivity biological layers typically introduces capacitive loading that lowers a fundamental frequency, the severe electromagnetic damping of the human tissue in this scenario suppressed the lower portion of the band (near 2.79 GHz). Consequently, the higher-order secondary resonance facilitated by the DPS and interdigital slots coupled more efficiently to the loaded environment and emerged as the primary matching point. Despite this complex near-field interaction, the $S_{11}$ remained well below the −10 dB threshold across a 910 MHz span. This resilience is mathematically attributed to the drastic reduction of the loaded Q-factor from approximately 40 to 1.86. Such a low Q-factor ensures that the antenna is less sensitive to the reactive loading of biological media, making it an ideal candidate for continuous monitoring where the sensing environment is dynamic rather than static.

To quantitatively assess this environmental robustness, the detuning sensitivity of the antenna under varying air gap distances is summarised in Table 6 and corresponding Figure 17. The data confirm that even under a severe 5.0 mm gap fluctuation, the antenna maintains a functional wideband response of 0.83 GHz, ensuring uninterrupted telemetry.

### 7.4. Anatomical and Dielectric Sensitivity Analysis

To ensure robust operation across diverse WBAN deployment scenarios, the antenna’s sensitivity to varying anatomical locations and tissue properties was evaluated. Body locations such as the arm, chest, and abdomen present varying thicknesses of skin and subcutaneous fat. Because the proposed design relies on a wide 43.7% fractional bandwidth matching buffer, anatomical variations primarily induce a shift in the primary resonance rather than causing complete impedance mismatch. A localised sensitivity analysis varying the tissue phantom’s relative permittivity ($\epsilon_{r}$) by ±10% was conducted to simulate fluctuating hydration levels and patient-specific dielectric properties. The simulated results demonstrate that under a +10% increase in tissue permittivity, the primary resonance shifted to 3.18 GHz, while a −10% decrease in permittivity shifted the resonance to 3.23 GHz. In both extreme scenarios, the antenna comprehensively maintained its −10 dB impedance matching across the primary target sub-6 GHz telemetry bands, confirming that the defected patch structure provides exceptional detuning immunity across dynamic on-body deployment sites, as seen in Table 7 and Figure 18.

## 8. Experimental Validations

### 8.1. Prototype Fabrication

The antenna prototype was fabricated on Rogers RO3006 substrate (h = 0.8 mm) using an LPKF ProtoMat S103 CNC milling machine with ±0.025 mm dimensional accuracy. The conducting layers were 1 oz (35 μm) copper. A 50 Ω SMA female end-launch connector (Amphenol 132291) was soldered to the microstrip feed line.

The excellent agreement between the measured and simulated, particularly the precise capture of the merged resonance loop on the Smith chart, serves as a proxy validation for the antenna’s modal behaviour. Consequently, the simulated radiation patterns and high radiation efficiency (>90%) are considered highly representative of the physical prototype’s performance

Photographs of the fabricated prototype are shown in Figure 19. Measured physical dimensions are 25.05 mm × 37.02 mm × 0.8 mm (with/without SMA connector), within ±0.1 mm of design specifications.

### 8.2. Measurement Setup

The reflection coefficient was measured using a Keysight PNA-X N5247B vector network analyser, calibrated using an electronic calibration module (ECal N4694-60001) over 1–5.5 GHz with 1601 points. Measurements were performed at 23 °C ± 2 °C.

### 8.3. Measured Reflection Coefficient

The measured S_11_ response is presented in Figure 20 and compared with simulation in Table 8.

Key measured results are as follows:

- Resonant frequency: 2.84 GHz (simulation: 2.79 GHz, deviation: +1.8%)
- Return loss at resonance: 26.8 dB (simulation: 29.4 dB)
- Lower −10 dB frequency: 2.54 GHz (simulation: 2.51 GHz, deviation: +1.2%)
- Upper −10 dB frequency: 3.96 GHz (simulation: 4.01 GHz, deviation: −1.2%)
- Measured −10 dB bandwidth: 1.42 GHz
- Measured fractional bandwidth: 43.7% (referenced to 3.25 GHz).

The measured results in Table 8 show good agreement with the simulation. The observed deviations are attributed to the following factors:

- Substrate permittivity tolerance: RO3006 has εᵣ = 6.5 ± 0.15 (±2.3% tolerance), directly affecting resonant frequency.
- Fabrication tolerances: CNC milling accuracy of ±0.025 mm affects slot dimensions and the inter-finger gap.
- SMA connector parasitic effects: Manual soldering variations introduce additional reactance not perfectly captured in the simulation.
- Copper surface roughness: At higher frequencies, surface roughness increases effective resistance;
- Substrate thickness variation: Nominal ±0.05 mm tolerance affects impedance.

The 5.3% reduction in measured bandwidth compared to simulation is consistent with the cumulative effect of these tolerances, which typically cause slight bandwidth narrowing in high-Q structures.

To quantitatively assess the impact of these real-world manufacturing variations, a Monte Carlo yield analysis was performed prior to fabrication. The statistical simulation incorporated a uniform distribution variance of ±0.025 mm on critical milled dimensions specifically the inter-finger gaps and slot widths alongside the manufacturer-specified ±0.15 tolerance in the substrate’s relative permittivity. Across the randomized iterations, the analysis demonstrated a high production yield, with the antenna consistently maintaining a −10 dB impedance bandwidth covering the primary target bands. While these combined statistical tolerances logically account for the 5.3% reduction in the measured bandwidth and the minor 1.8% frequency shift observed in the physical prototype, the Monte Carlo results ultimately confirm that the design’s wideband “matching buffer” provides robust resilience against standard fabrication anomalies, validating its suitability for reliable mass production.

### 8.4. Measured Radiation Performance

While full 3D radiation pattern measurements in an anechoic chamber could not be obtained for this study, the precise agreement between the measured and simulated $\left|S_{11}\right|$ response serves as a highly reliable proxy for the antenna’s modal behaviour. Specifically, the accurate experimental replication of the merged resonance loop on the Smith chart provides strong indirect evidence that the underlying modal behaviour, and by extension, the surface current distribution, aligns closely with the simulated model. Because the radiation characteristics are directly derived from these surface currents, the simulated quasi-omnidirectional radiation patterns and high radiation efficiency (>90%) are considered highly representative of the physical prototype’s performance. Comprehensive far-field characterisation remains a primary objective for future progression of this telemetry node.

## 9. Performance Comparison

A comparison of the proposed antenna with recently published wideband compact microstrip patch antennas is presented in Table 9.

While coplanar waveguide (CPW) and slot antennas can achieve fractional bandwidths exceeding 100%, their bidirectional radiation patterns make them highly susceptible to severe detuning and signal absorption when placed in proximity to human tissue. In contrast, the proposed microstrip patch design utilises a ground plane to shield the biological tissue from backward radiation. Achieving a 43.7% measured fractional bandwidth on a highly miniaturised, high-permittivity ($\epsilon_{r}=6.5$) patch topology represents a significant advancement for directional, on-body WBAN deployment.

Key observations:

- Fractional bandwidth: The proposed antenna achieved the highest fractional bandwidth (43.7% measured) among comparable single-layer designs, exceeding the nearest single-layer competitor by 3.6 percentage points;
- Compactness: With a 925 mm^2^ footprint, the proposed antenna is among the most compact, particularly considering the achieved bandwidth;
- High-permittivity substrate: The proposed design demonstrates that wideband performance is achievable on high-permittivity substrates (εᵣ = 6.5) through proper slot and defect engineering, whereas most comparable designs use lower-permittivity materials;
- Gain trade-off: The peak in-band gain of 3.2 dBi is moderate compared to designs on thicker, low-permittivity substrates. This is an expected trade-off with high-permittivity, thin substrates that inherently limit radiation aperture efficiency;
- Design simplicity: Unlike stacked patches or complex metasurface designs, the proposed antenna uses a single-layer topology, simplifying fabrication;
- Gain-bandwidth product (GBP): To contextualise the moderate peak gain of 3.2 dBi, the GBP (FBW×linear gain) was evaluated. The proposed antenna achieved a highly competitive GBP of 91.3. While designs utilising thick, low-permittivity substrates (e.g., [14,30]) inherently achieve higher efficiencies and GBP scores, the proposed design outperforms several physically larger antennas operating on lower permittivity substrates (e.g., [17,29,44]). This confirms that the proposed slot engineering technique provides an optimal trade-off, maximizing bandwidth without severely degrading the radiation mechanisms fundamental to high-permittivity miniaturized patches.

## 10. Conclusions

A compact wideband circular microstrip patch antenna incorporating bilateral interdigital capacitive slots and a central defected patch structure has been systematically designed, analysed, and experimentally validated. The antenna is realised on a Rogers RO3006 substrate (εᵣ = 6.5, h = 0.8 mm) with a compact footprint of 25 × 36.96 mm^2^.

The five-step design evolution clearly demonstrated the progressive bandwidth enhancement, as follows:

- ANT1 (baseline): 10% fractional bandwidth
- ANT2 (unilateral slots): 17% fractional bandwidth
- ANT3 (bilateral slots): 27% fractional bandwidth
- ANT4 (DPS addition): 37% fractional bandwidth
- ANT5 (optimized): 46% simulated, 43.7% measured fractional bandwidth

A comprehensive parametric study established the sensitivity of key design parameters, providing guidelines for adaptation to other frequency bands. An equivalent circuit model was developed and validated against full-wave simulation, demonstrating that the loaded Q-factor is reduced from approximately 40 (basic patch) to approximately 1.86 (final design).

The fabricated prototype demonstrated good agreement with simulation, with results as follows:

- Measured resonant frequency: 2.84 GHz (1.8% deviation from simulation)
- Measured bandwidth: 2.54–3.96 GHz (1.42 GHz, 43.7% fractional bandwidth)
- Simulated peak in-band gain: 3.2 dBi at 3.8 GHz
- Simulated radiation efficiency: >90% across operating band

The proposed antenna is suitable for wireless applications, including the following specifications:

- 2.4 GHz ISM band
- LTE bands 7 (2.5–2.69 GHz), 38 (2.57–2.62 GHz), 41 (2.496–2.69 GHz)
- 5G NR bands n77 (3.3–4.2 GHz, partial coverage) and n78 (3.3–3.8 GHz)
- WiMAX 2.5/3.5 GHz bands

The proposed antenna demonstrates a robust solution for biomedical telemetry and sensing. Its high efficiency (>90%) and compact single-layer topology satisfy the requirements for low-profile telemetry nodes, while successfully maintaining functional on-body radiation efficiency (8.4%) under severe tissue loading conditions.

Finally, it should be noted that full radiation pattern measurements in an anechoic chamber were not available for this study; addressing this limitation through comprehensive far-field characterisation forms a primary objective for future work. As WBAN systems frequently require multi-node deployment, future research will investigate the antenna’s integration into multiple-input multiple-output (MIMO) array configurations. While comprehensive MIMO analysis and envelope correlation coefficient (ECC) evaluation are beyond the scope of this single-element design study, the wideband nature and localised surface current distributions of the proposed antenna make it highly suitable for high-isolation array integration, which we plan to investigate in future work. Additionally, subsequent research will leverage the antenna’s robust wideband ‘matching buffer,’ which effectively minimises detuning anomalies and ensures consistent data transmission in dynamic on-body environments. Building upon this stable hardware foundation, we are currently expanding our dataset collection across the 3–6 GHz spectrum and developing the required machine learning algorithms. Ultimately, integrating this high-quality spectral data with physics-guided machine learning models will pave the way for highly accurate, automated classification of complex bone fracture states.

## Figures and Tables

**Figure 1:**
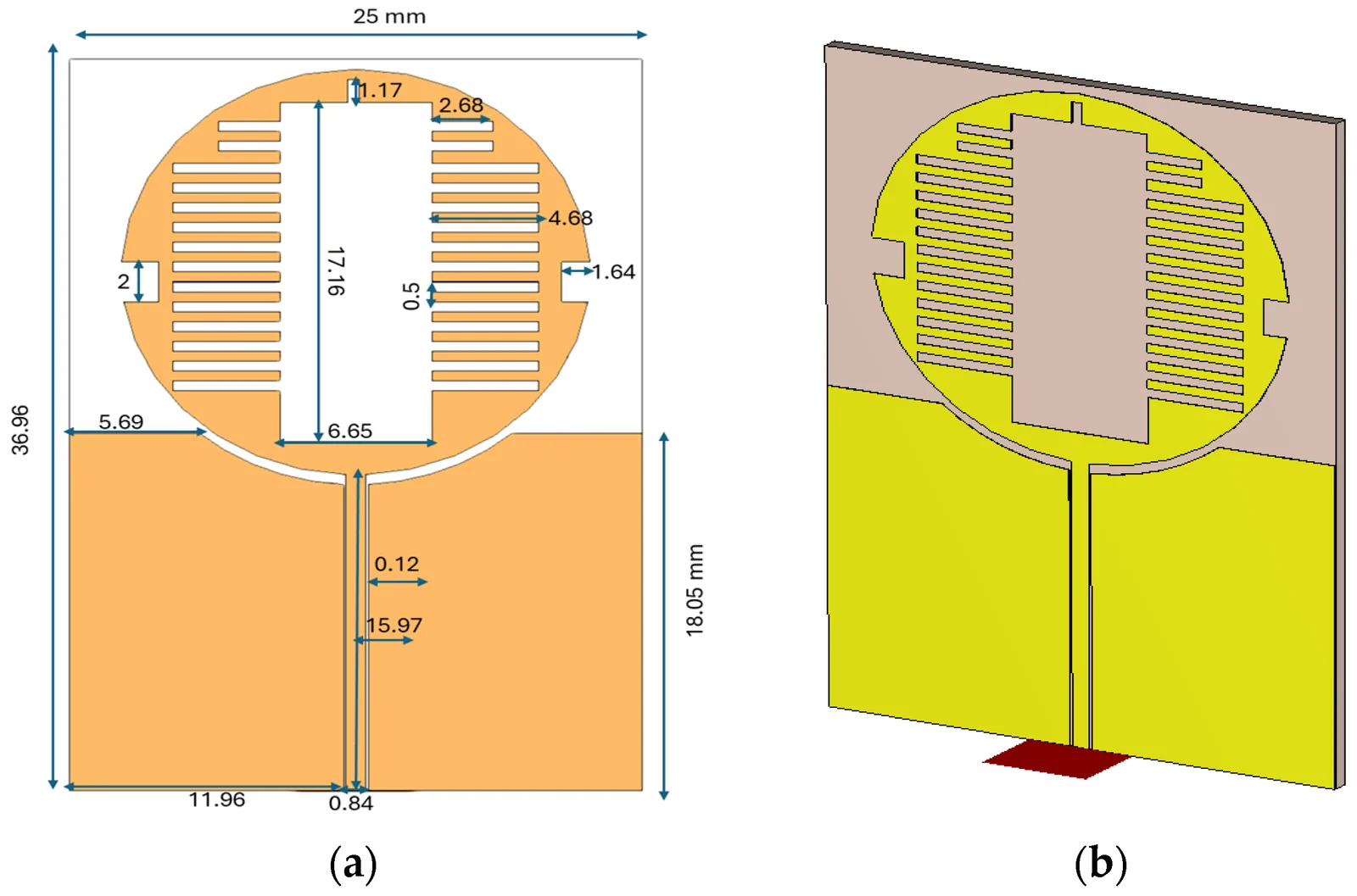
(**a**) Geometry and dimensional details of the proposed antenna (ANT5): top view showing circular patch with bilateral interdigital slots, central DPS, microstrip feed line, and partial ground plane (all dimensions in mm); (**b**) side view of the proposed antenna with port.

**Figure 2:**
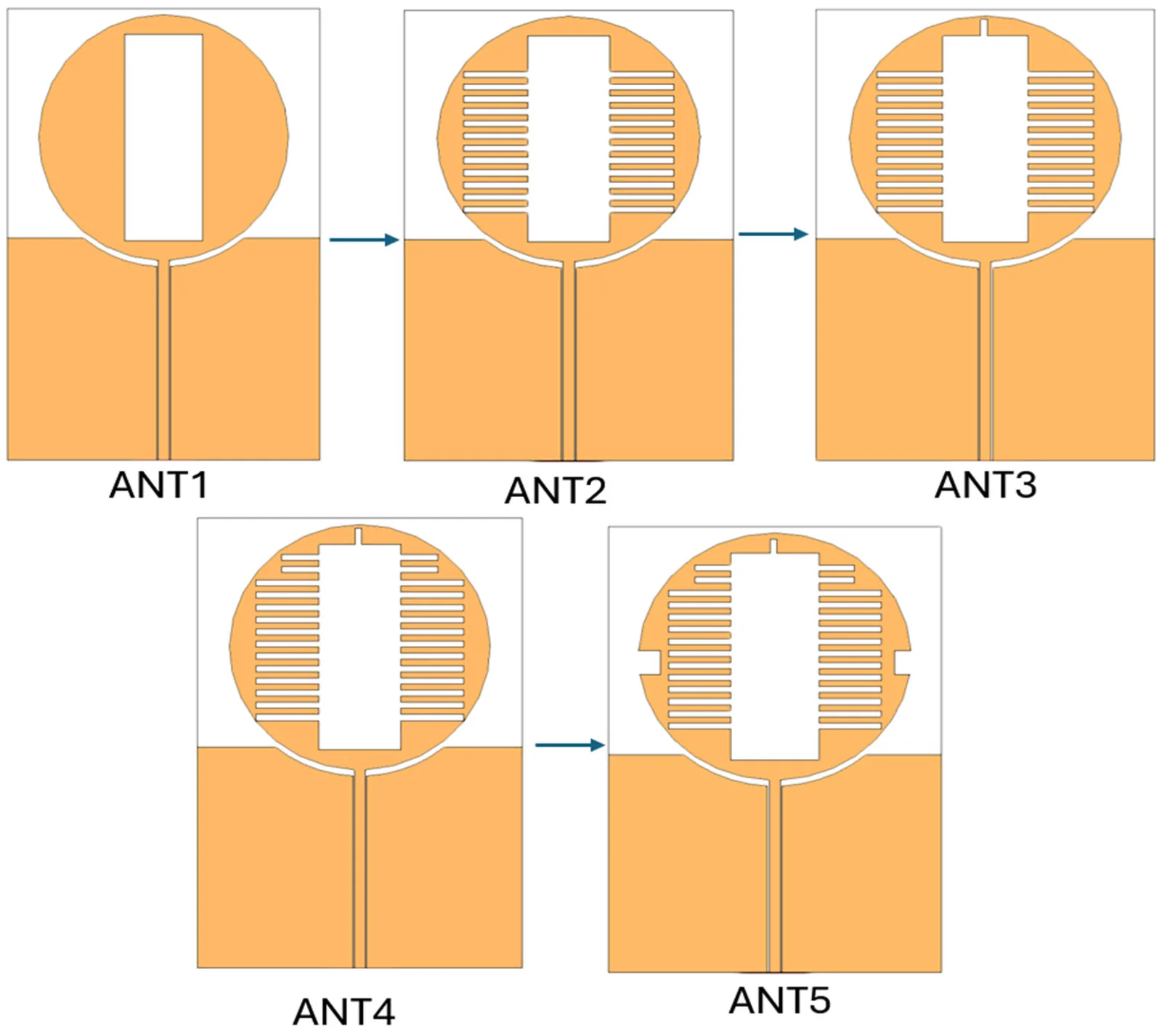
Design evolution stages of the proposed antenna: ANT1 (reference patch with single slot), ANT2 (left-side interdigital slots), ANT3 (bilateral interdigital slots), ANT4 (addition of central DPS), and ANT5 (optimized final design).

**Figure 3:**
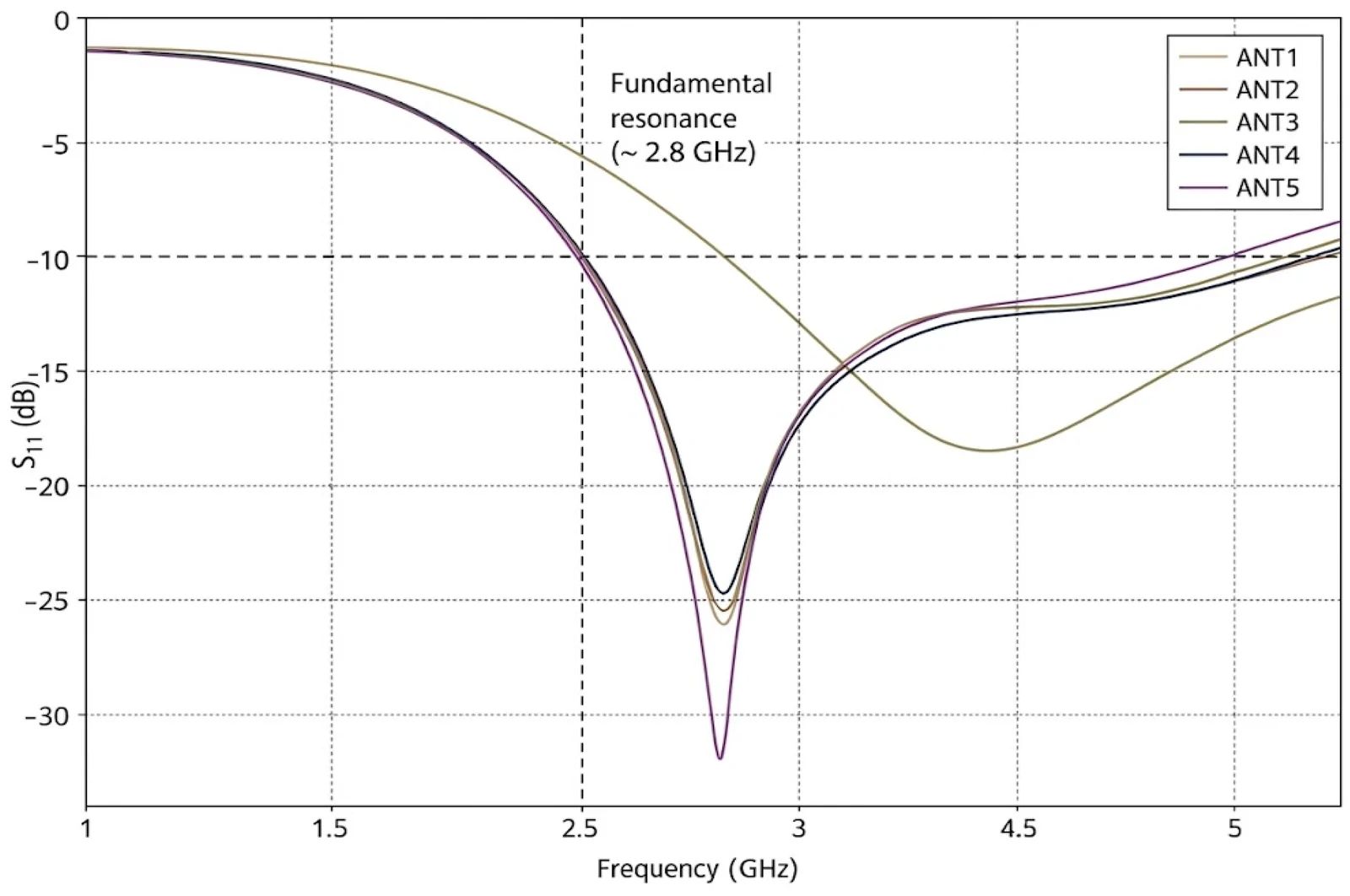
Simulated |S_11_| responses of antenna variants ANT1–ANT5, demonstrating progressive bandwidth enhancement through systematic integration of interdigital slots and DPS.

**Figure 4:**
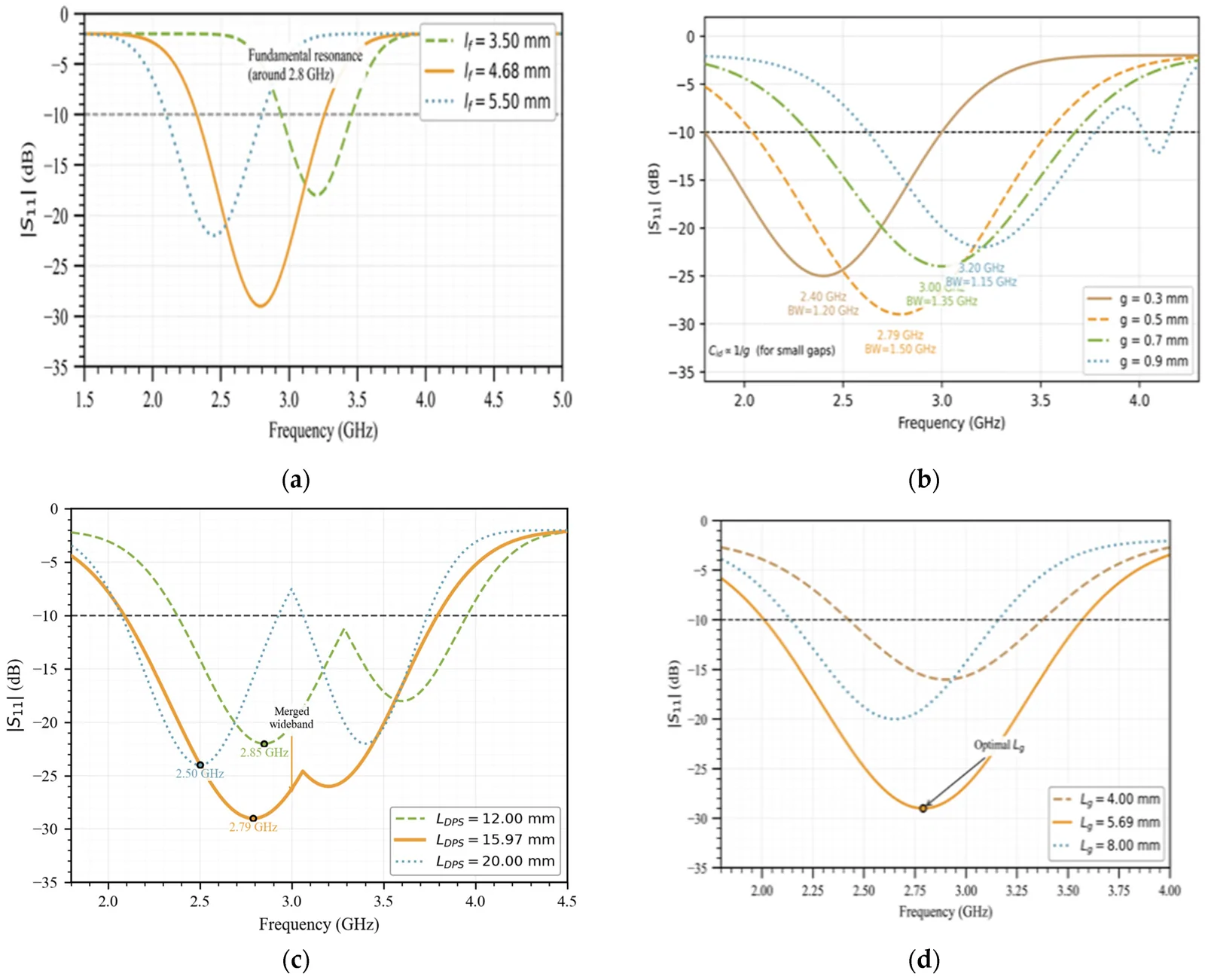
Simulated |S_11_| characteristics for parametric analysis of key antenna dimensions: (**a**) varying slot finger length *l_f_*; (**b**) varying inter-finger gap *g*; (**c**) varying *DPS* length *L_DPS_*; and (**d**) varying partial ground plane length *Lg*.

**Figure 5:**
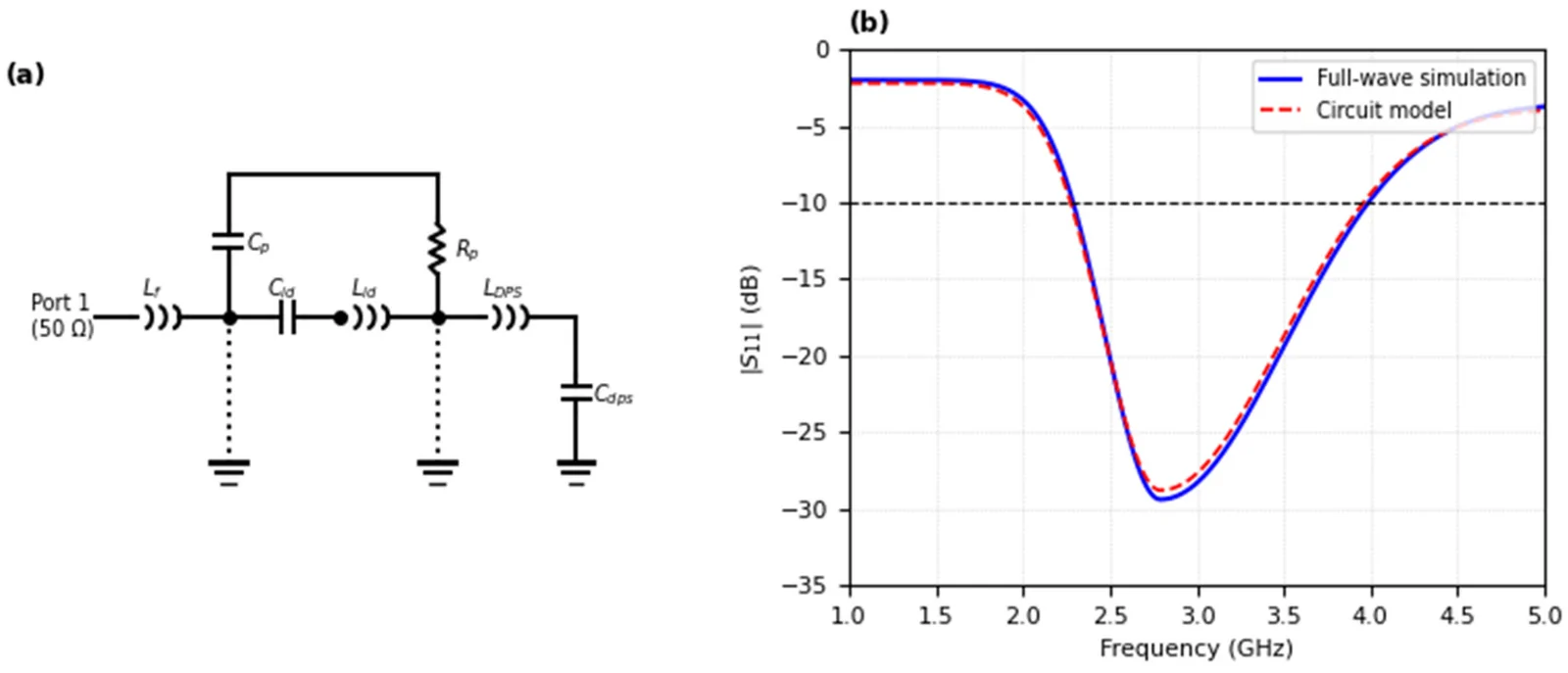
Equivalent circuit modelling of the proposed antenna. (**a**) Lumped-element equivalent circuit representation showing the feed inductance ($L_{f}$), patch capacitance ($C_{p}$), interdigital capacitance ($C_{id}$), slot inductance ($L_{id}$), radiation resistance ($R_{p}$), DPS inductance ($L_{DPS}$), and ground capacitance ($C_{dps}$). (**b**) Comparison of the reflection coefficient ($|S_{11}|$) obtained from the proposed circuit model and full-wave electromagnetic simulation, demonstrating excellent agreement across the operating band.

**Figure 6:**
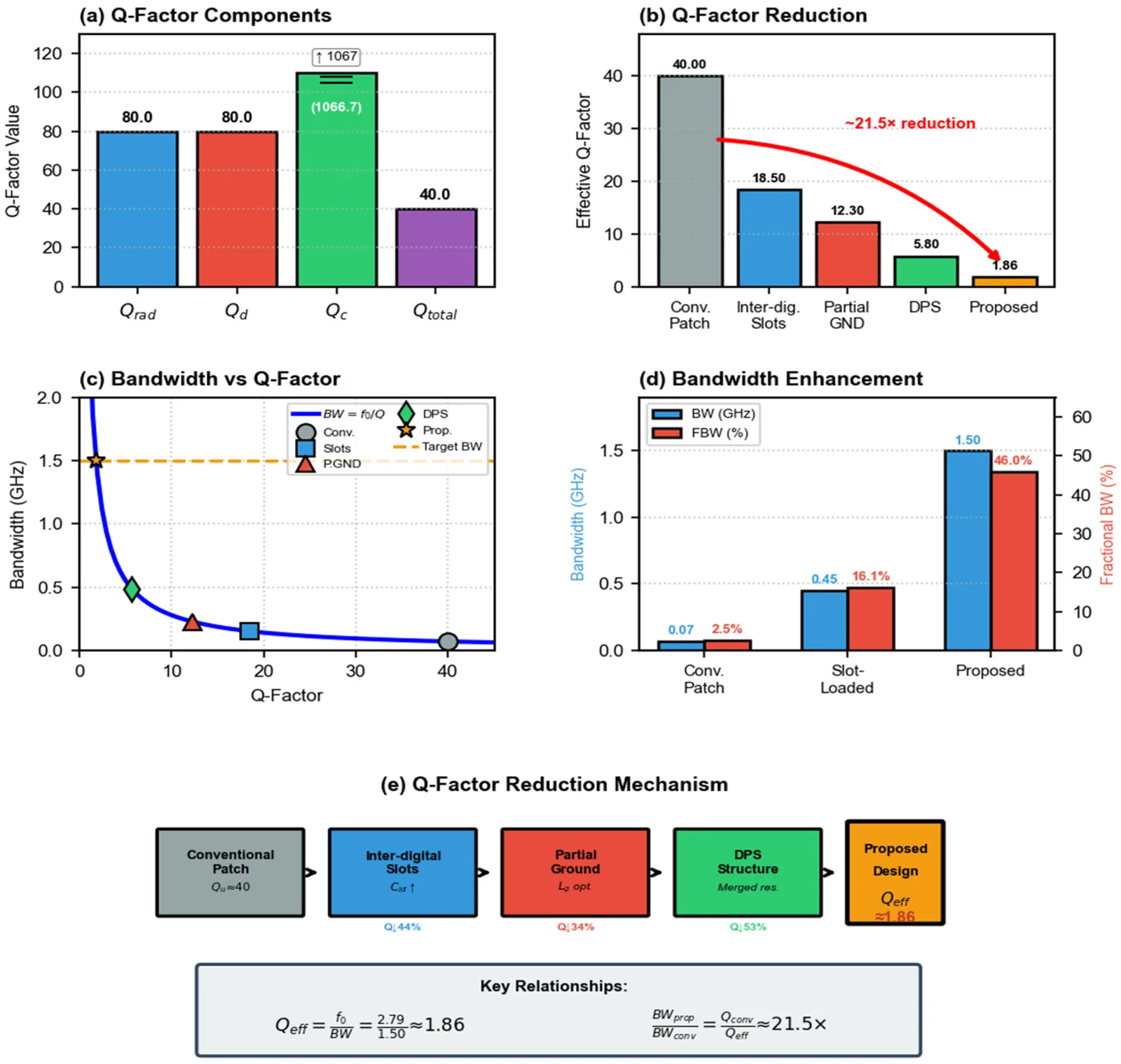
Q-factor analysis and bandwidth enhancement mechanism of the proposed antenna. (**a**) Q-Factor Components. (**b**) Progressive Q-factor reduction from approximately 40 (conventional) to 1.86 (proposed) achieved through interdigital slots, partial ground plane optimisation, and DPS structure. (**c**) Theoretical relationship between Q-factor and bandwidth showing the design trajectory. (**d**) Bar chart comparing absolute and fractional bandwidth enhancement across design stages, highlighting the progression to the final 46% fractional bandwidth. (**e**) Q-factor reduction mechanism outlining the key mathematical relationships demonstrating a 21.5× improvement.

**Figure 7:**
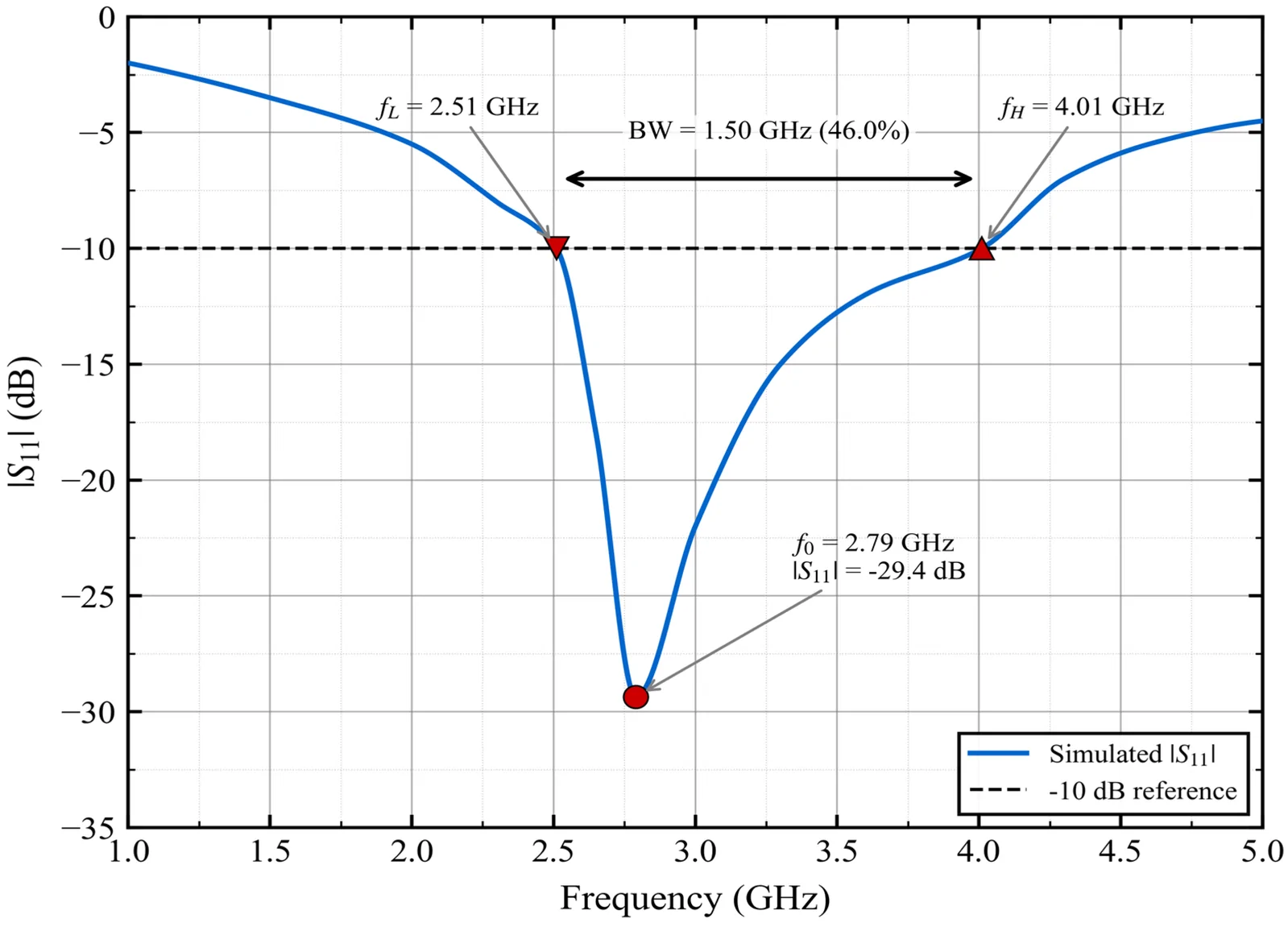
Simulated reflection coefficient ($|S_{11}|$) of the proposed interdigital slot-loaded circular patch antenna designed for wideband wireless sensing applications. The antenna demonstrates excellent impedance matching with a minimum $|S_{11}|$ of −29.4 dB at the resonant frequency of 2.79 GHz. The −10 dB impedance bandwidth spans from $f_{L}$ = 2.51 GHz to $f_{H}$ = 4.01 GHz, corresponding to 1.50 GHz absolute bandwidth and 46.0% fractional bandwidth. This wideband characteristic ensures robust operation under varying environmental conditions and provides sufficient spectral coverage for multi-parameter sensing.

**Figure 8:**
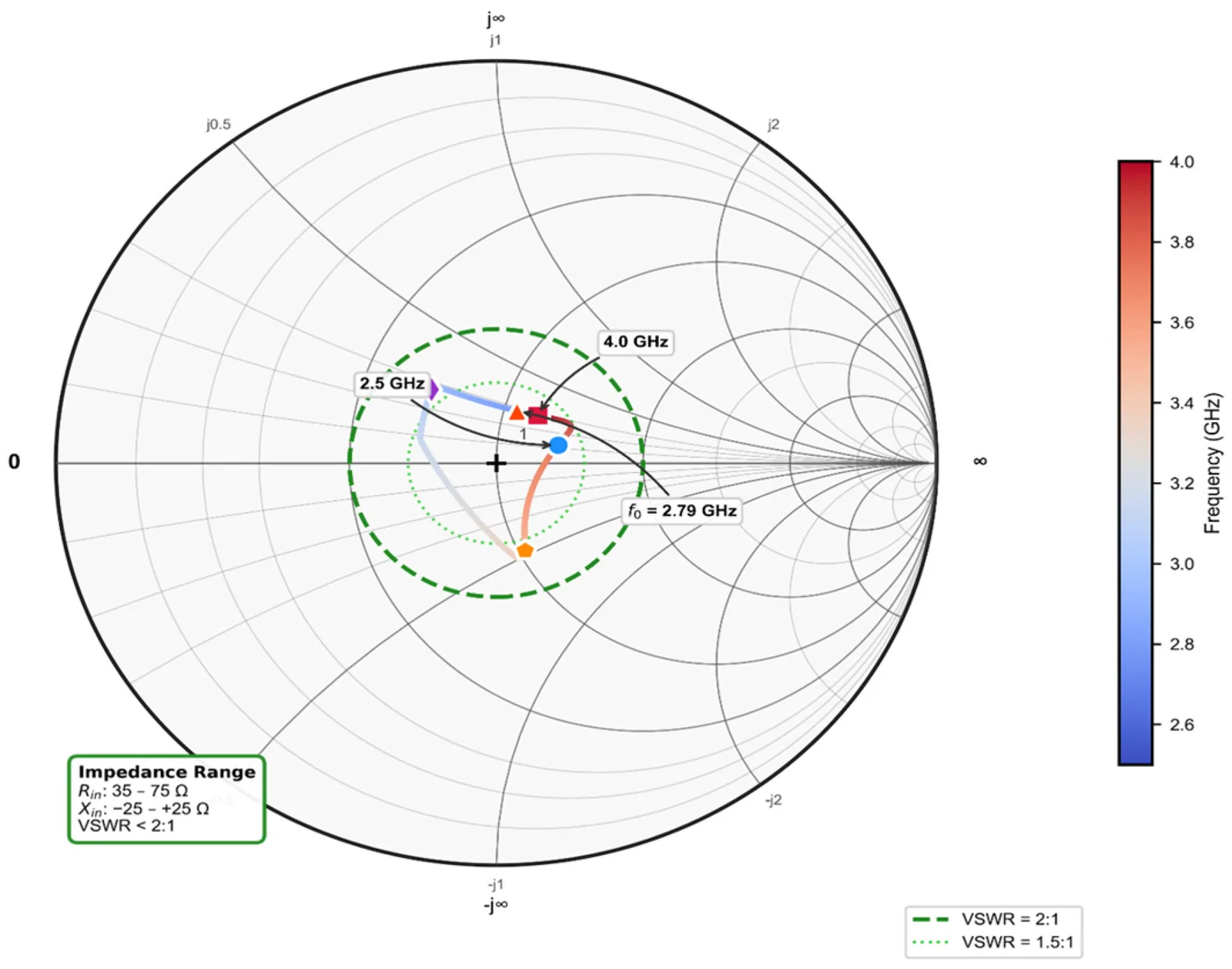
Simulated input impedance of the proposed antenna plotted on a Smith chart over the 2.5–4.0 GHz frequency range. The impedance locus forms a compact loop near the chart centre, indicating excellent impedance matching. Specific frequency markers are denoted by symbols: the orange triangle indicates the lower frequency bound (~2.5 GHz), the blue circle indicates the fundamental resonance ($f_{0}=2.79$ GHz), the red square indicates the upper frequency bound (~4.0 GHz), and the orange pentagon marks the point of maximum capacitive reactance within the wideband loop. The black cross (+) marks the perfect 50 Ω characteristic impedance centre.

**Figure 9:**
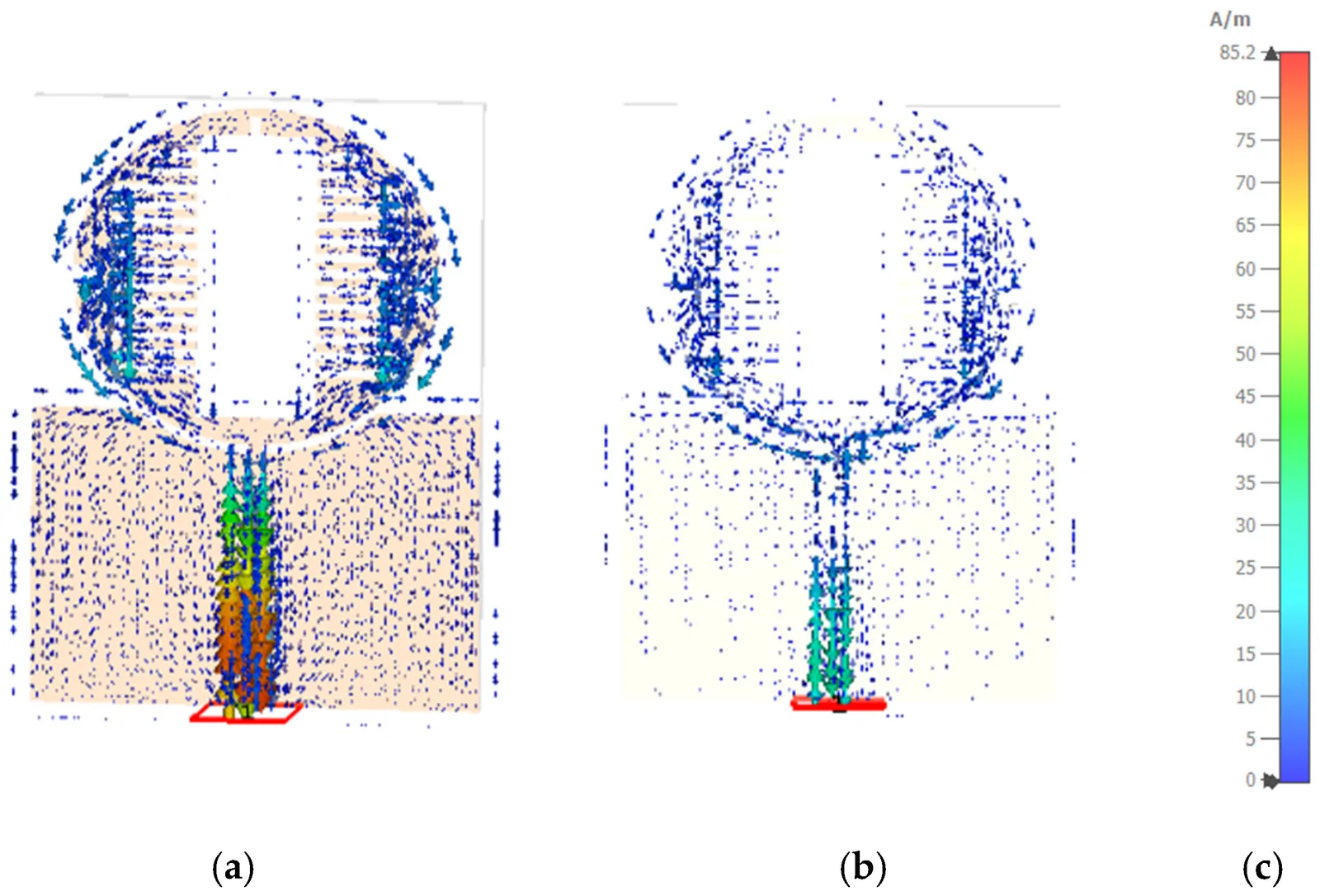
Simulated surface current distributions at (**a**) 2.8 GHz, highlighting strong capacitive coupling across the interdigital slots, (**b**) 3.8 GHz, demonstrating the inductive current concentrations along the central defected patch structure (DPS) and (**c**) The corresponding color scale indicating surface current density in A/m, where warmer colors (red/orange) denote regions of maximum current concentration and cooler colors (dark blue) denote regions of minimal current flow.

**Figure 10:**
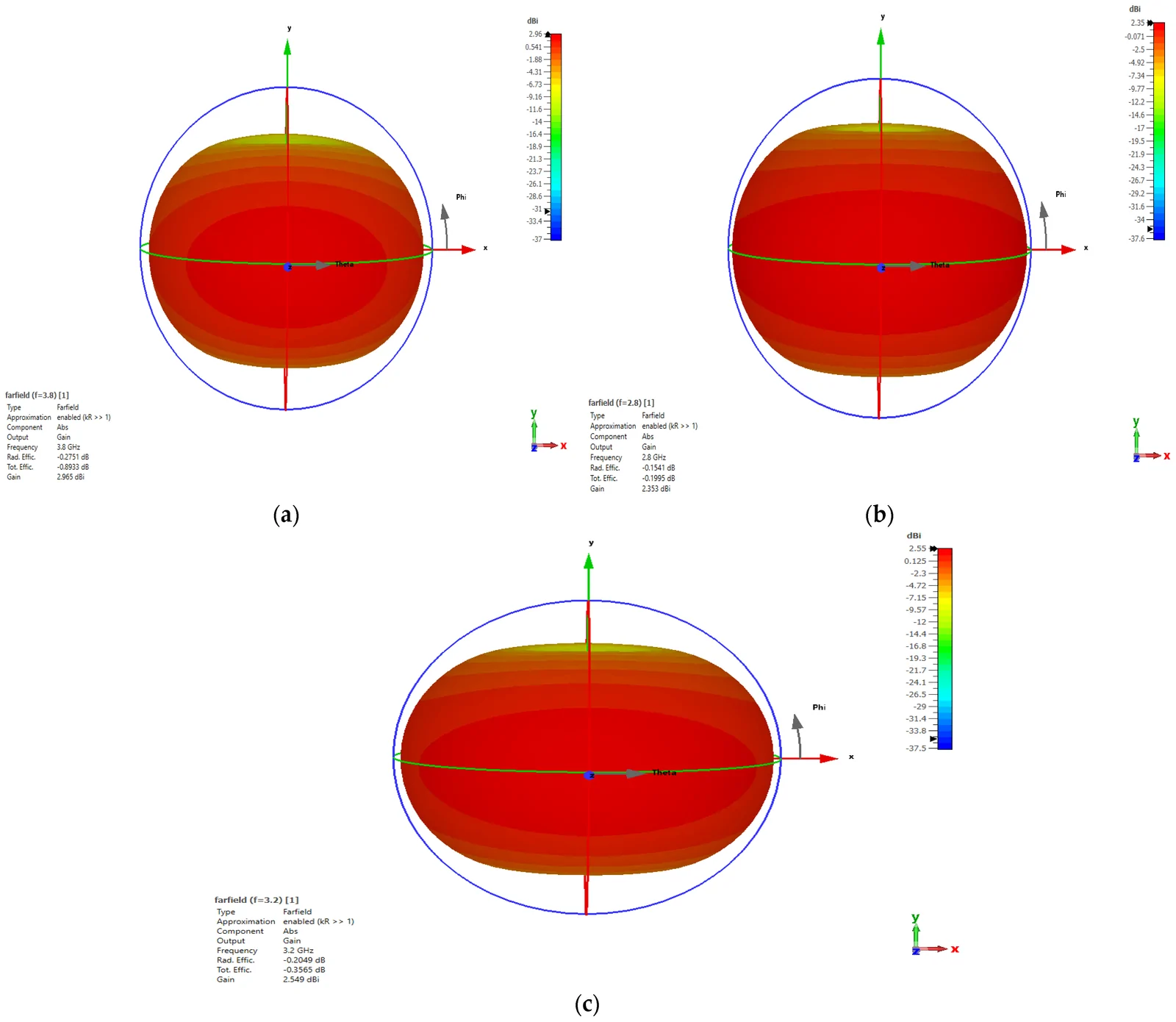
Simulated 3D far-field radiation patterns showing stable, quasi-omnidirectional broadside characteristics at (**a**) 2.8 GHz, (**b**) 3.2 GHz, and (**c**) 3.8 GHz. The patterns confirm consistent directional radiation suitable for wearable telemetry across the entire operating band.

**Figure 11:**
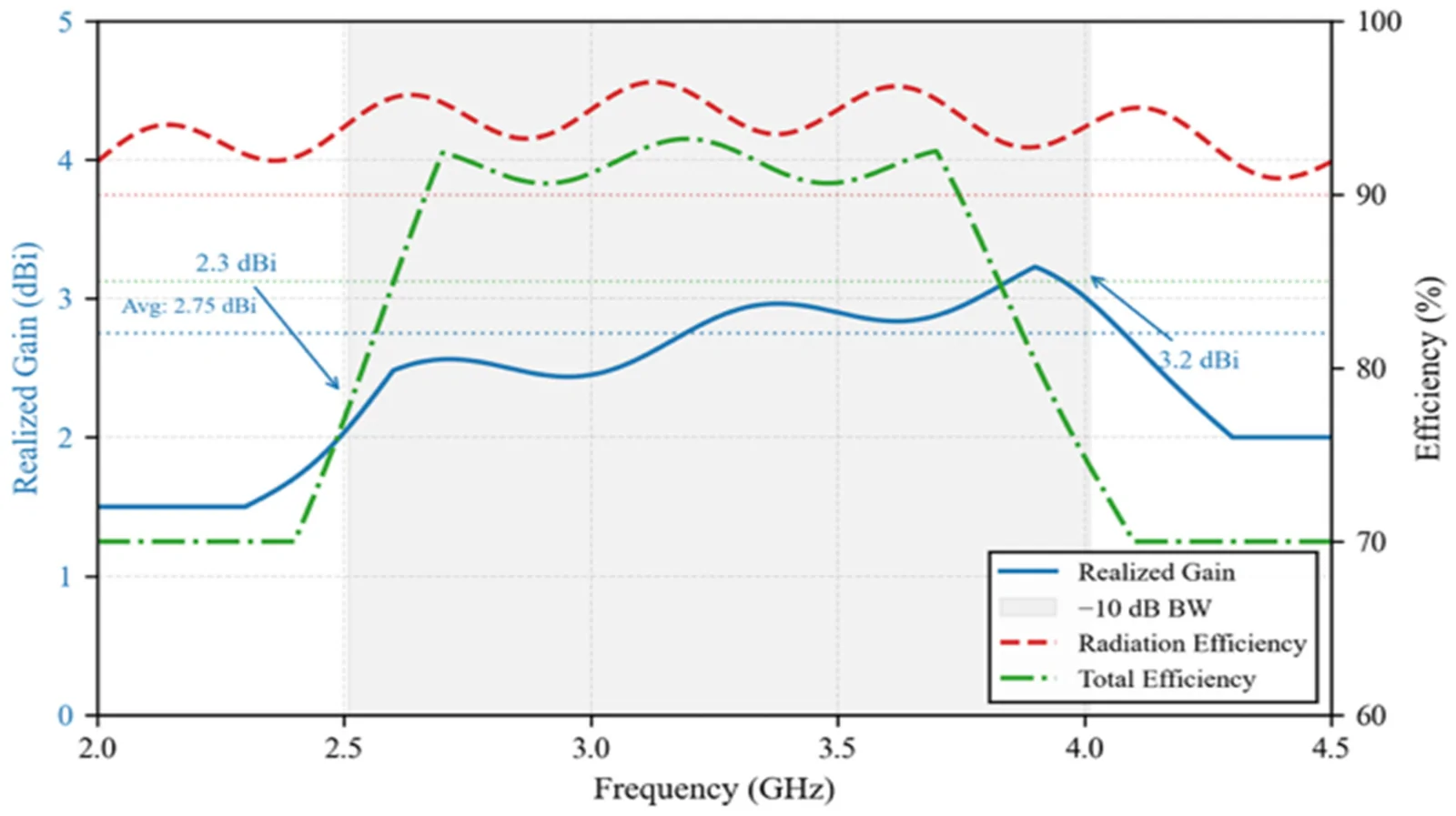
Simulated realised gain and efficiency of the proposed antenna. The realised gain varies from 2.3 dBi at 2.5 GHz to 3.2 dBi at 4.0 GHz with an average in-band gain of 2.75 dBi. Radiation efficiency exceeds 90% across the entire −10 dB bandwidth, while total efficiency remains above 85% within the 2.6–3.8 GHz range.

**Figure 12:**
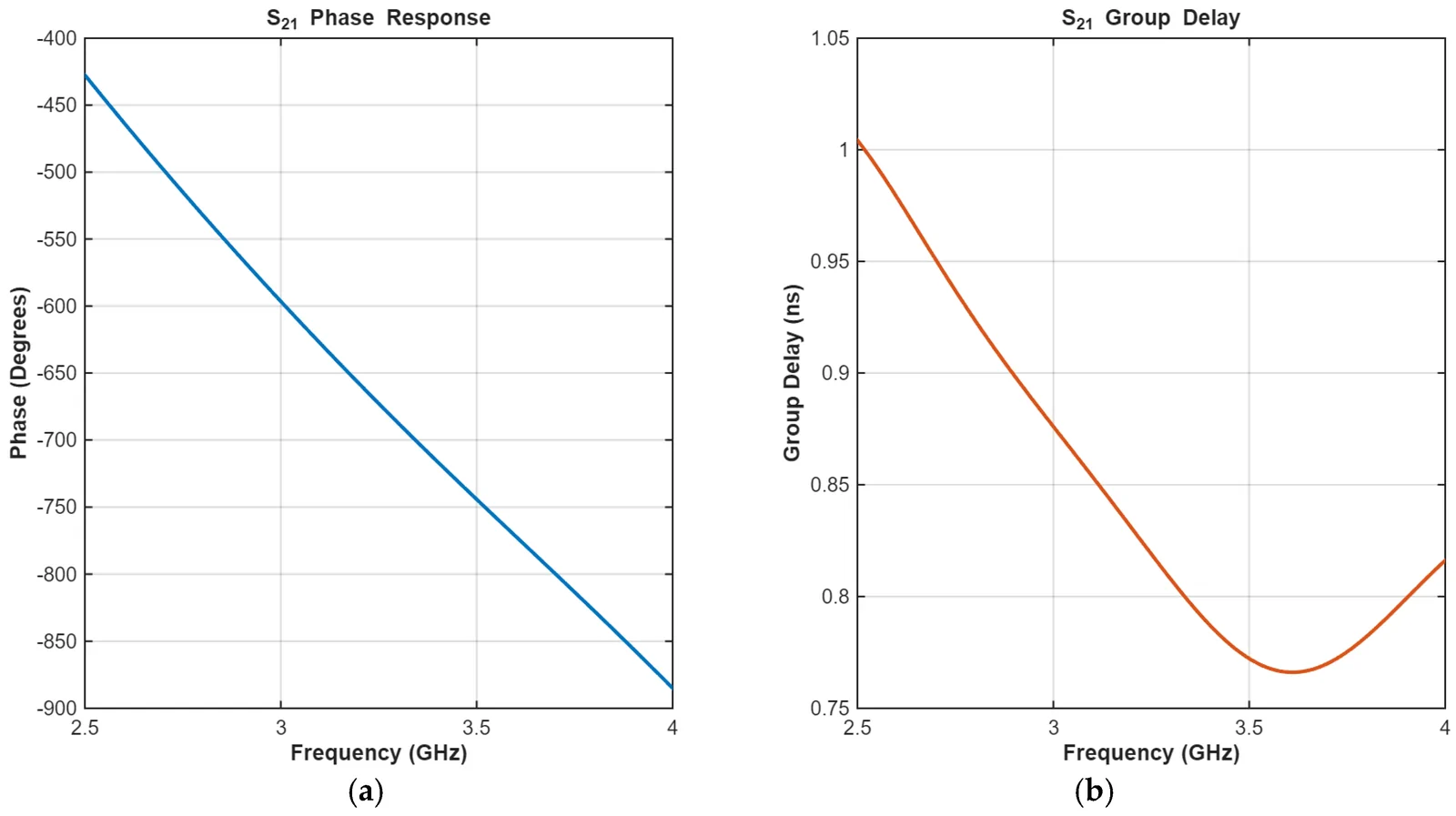
Simulated time-domain transmission characteristics of the proposed antenna in a face-to-face dual-antenna setup over the 2.5–4.0 GHz band. (**a**) The transmission phase response ($S_{21}$) demonstrates a highly linear profile. (**b**) The corresponding group delay indicates a maximum variation of less than 0.25 ns. These stable time-domain metrics confirm minimal phase distortion, ensuring signal integrity for high-data-rate, pulse-based WBAN telemetry.

**Figure 13:**
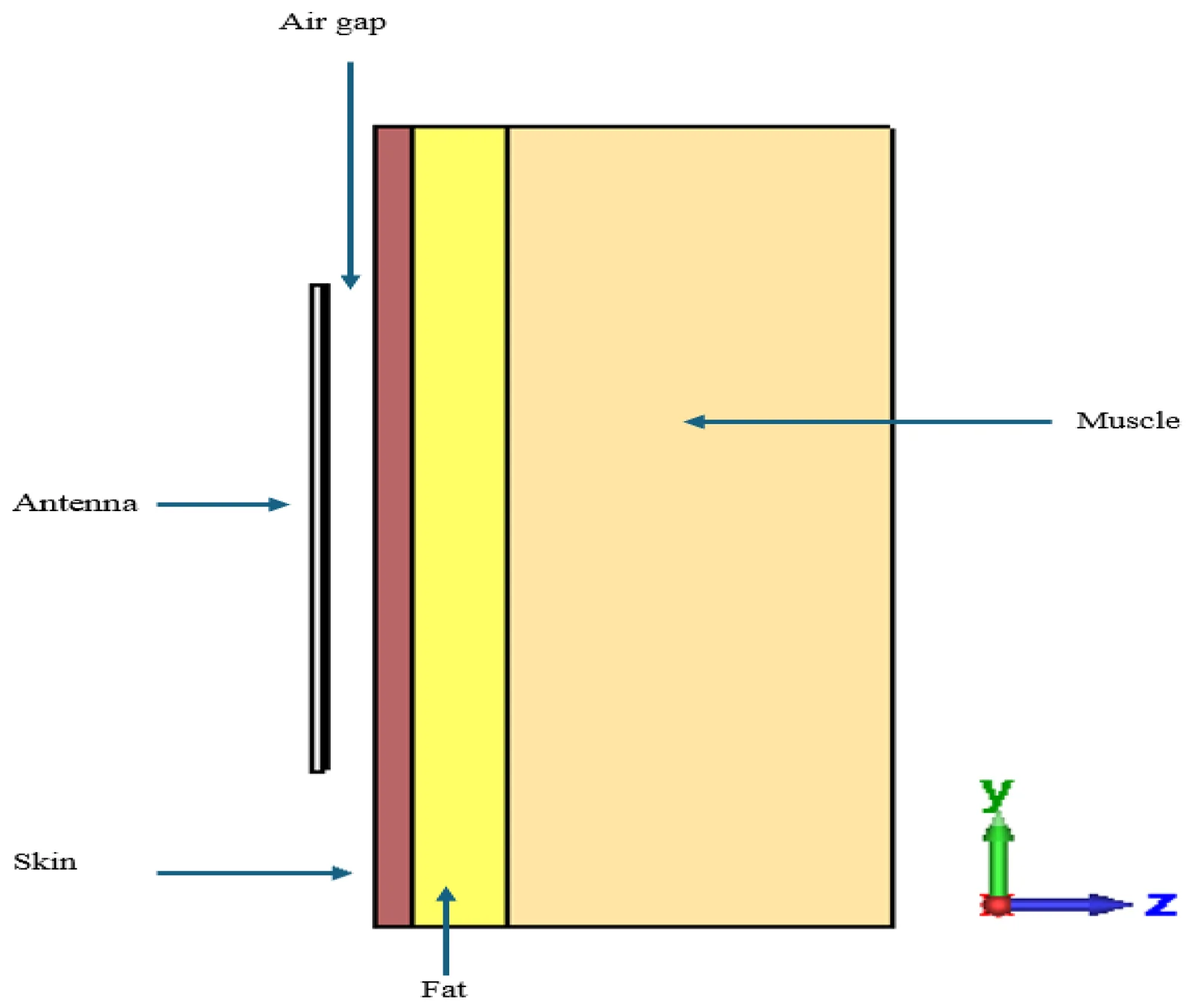
Schematic of the multi-layer biological tissue phantom simulation setup, detailing the antenna placement, the 2 mm air gap, and the stratified skin, fat, and muscle layers.

**Figure 14:**
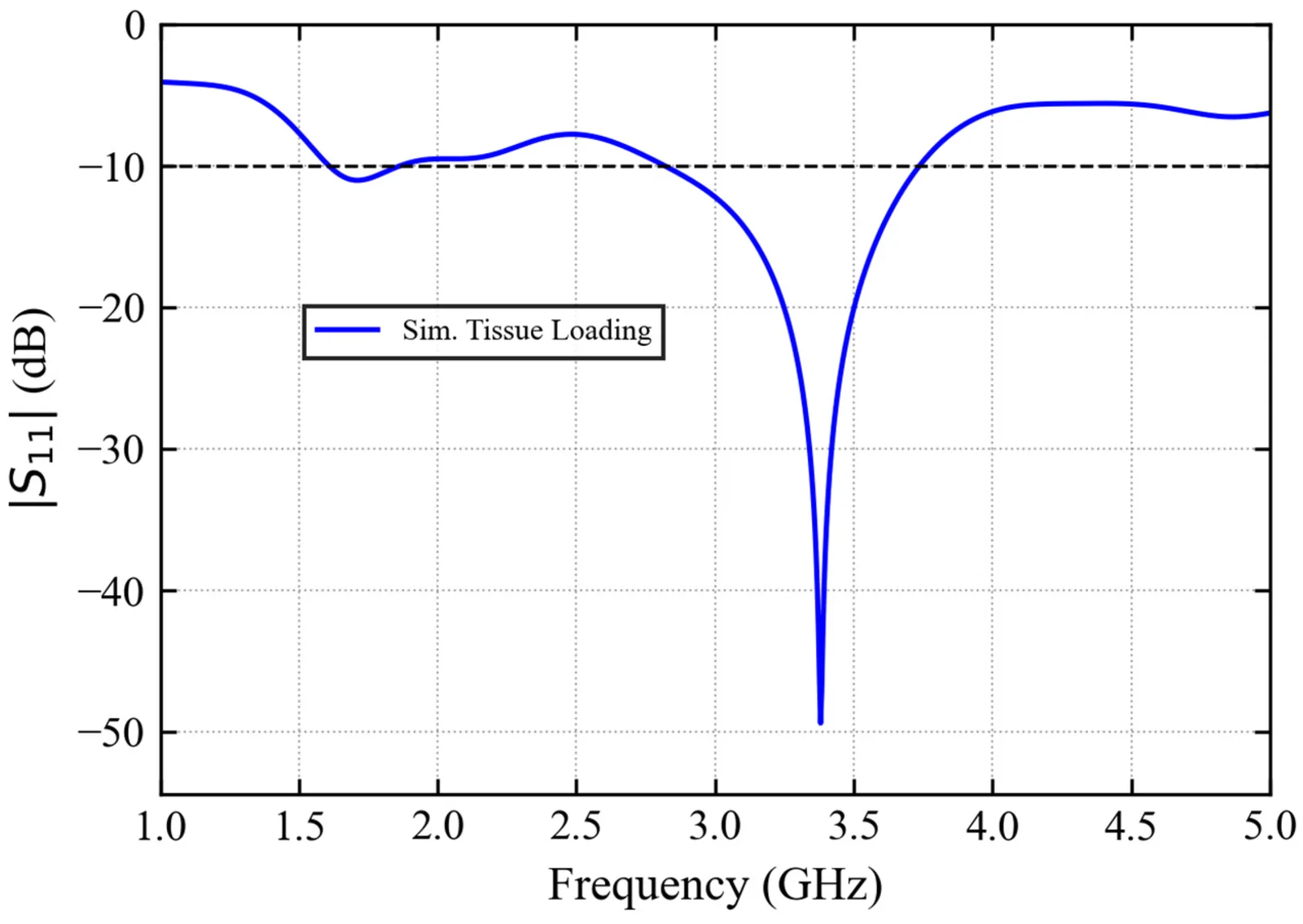
Simulated reflection coefficient (S_11_) of the proposed antenna loaded with the multi-layer tissue phantom. Due to the high permittivity of the biological media, the primary resonance shifts to ~3.38 GHz. However, the antenna maintains a robust −10 dB impedance bandwidth of 910 MHz (2.82–3.73 GHz), confirming improved robustness to severe detuning.

**Figure 15:**
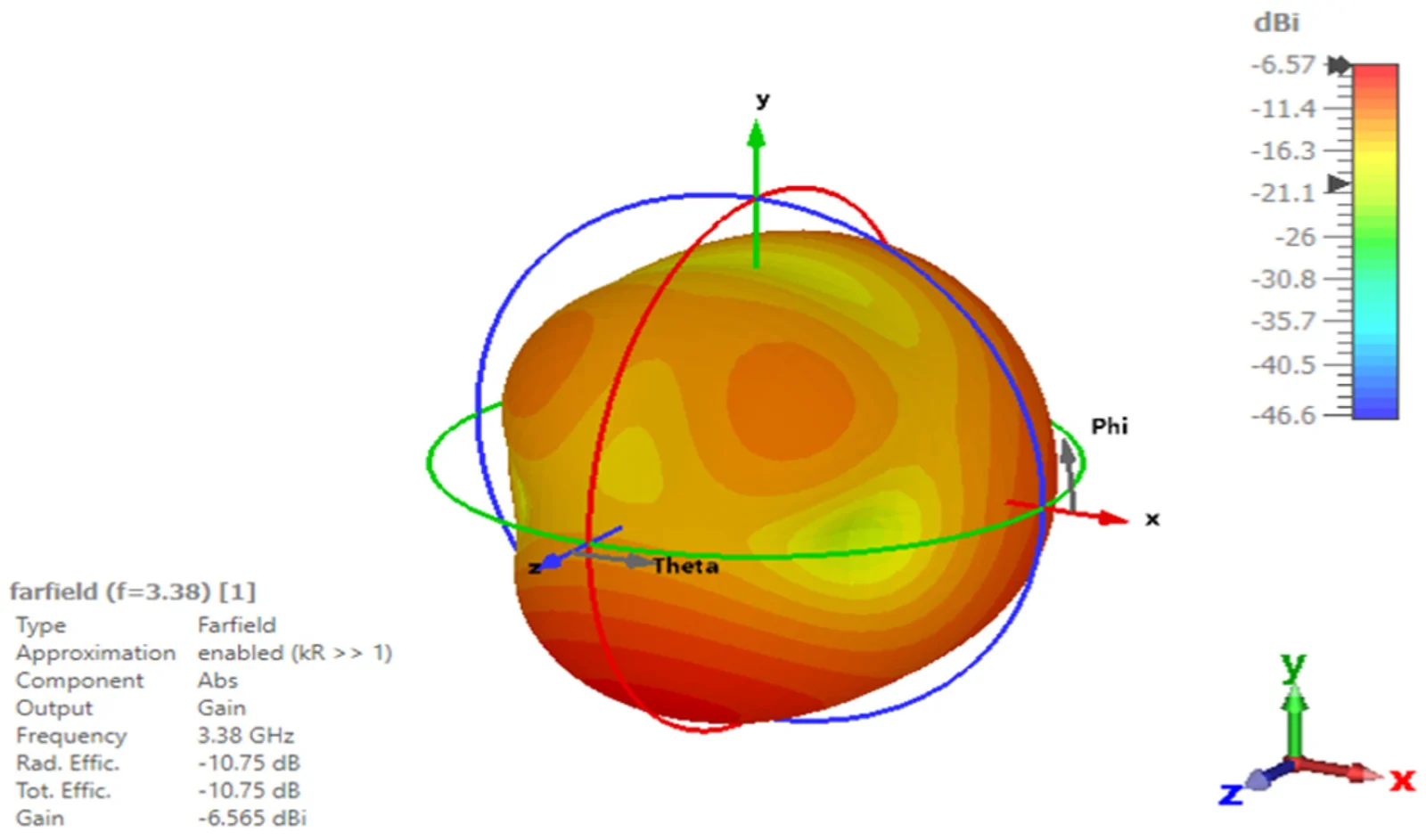
Simulated 3D far-field radiation pattern at the loaded resonant frequency of 3.38 GHz. As expected for wearable medical telemetry, proximity to highly lossy human tissue results in significant electromagnetic absorption. The antenna achieves a realised gain of −6.565 dBi and a radiation efficiency of −10.75 dB (8.4%), which is standard for on-body devices and sufficient for short-range Body Area Network (BAN) communication.

**Figure 16:**
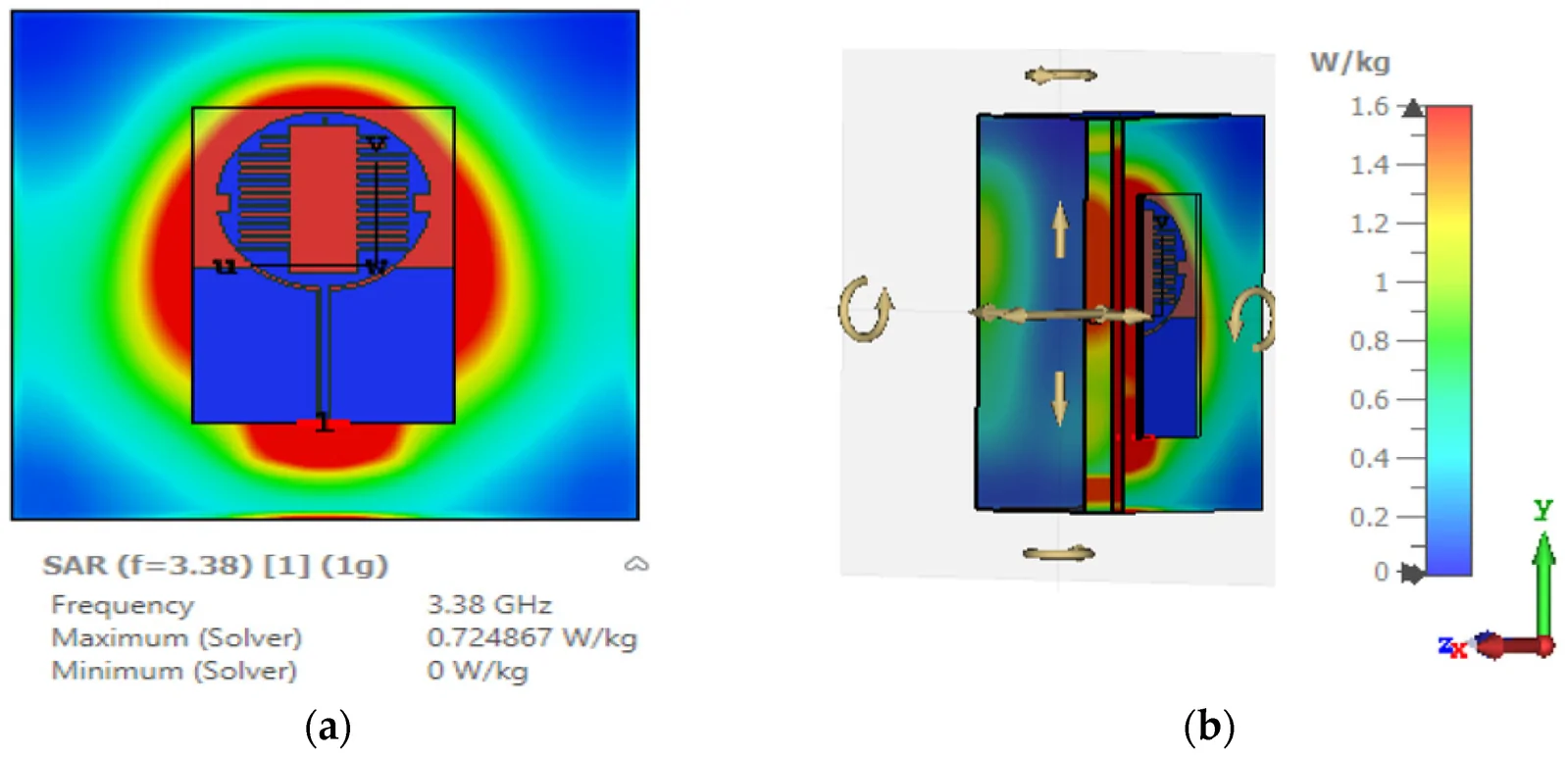
Simulated specific absorption rate (SAR) distribution at the loaded resonant frequency of 3.38 GHz over a 1 g tissue mass. (**a**) Top view and (**b**) cross-sectional view through the multi-layer tissue phantom (skin, fat, muscle). With an input power normalised to 10 mW, the maximum SAR is 0.724 W/kg, safely below the 1.6 W/kg FCC regulatory limit for wearable biomedical devices.

**Figure 17:**
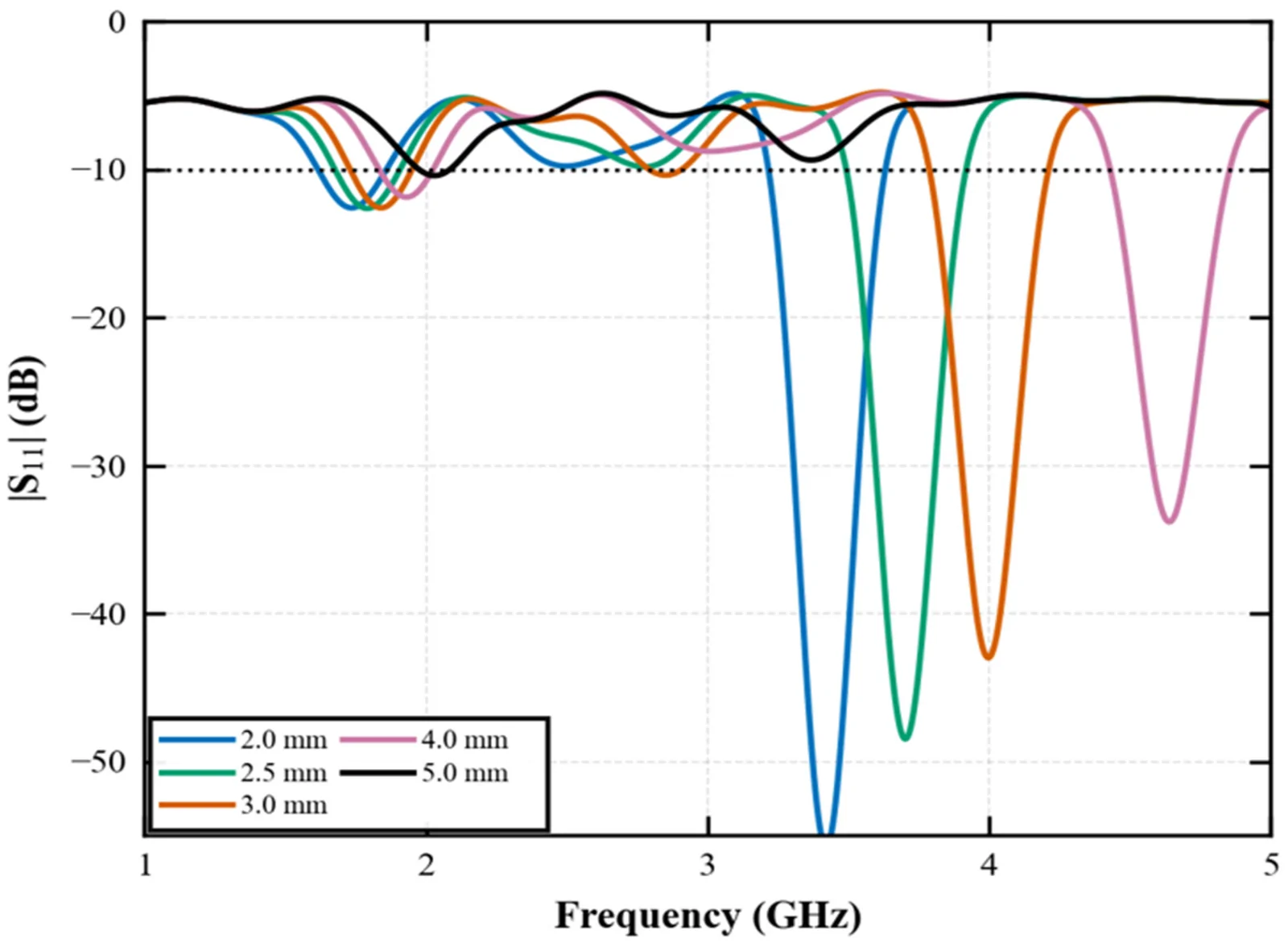
Simulated S_11_ characteristics of the proposed antenna under varying air gap distances from the tissue phantom. The results demonstrate the antenna’s robustness; despite frequency shifts as the distance increases from 2.0 mm to 5.0 mm, the wideband “matching buffer” ensures the antenna remains operational within the target frequency bands.

**Figure 18:**
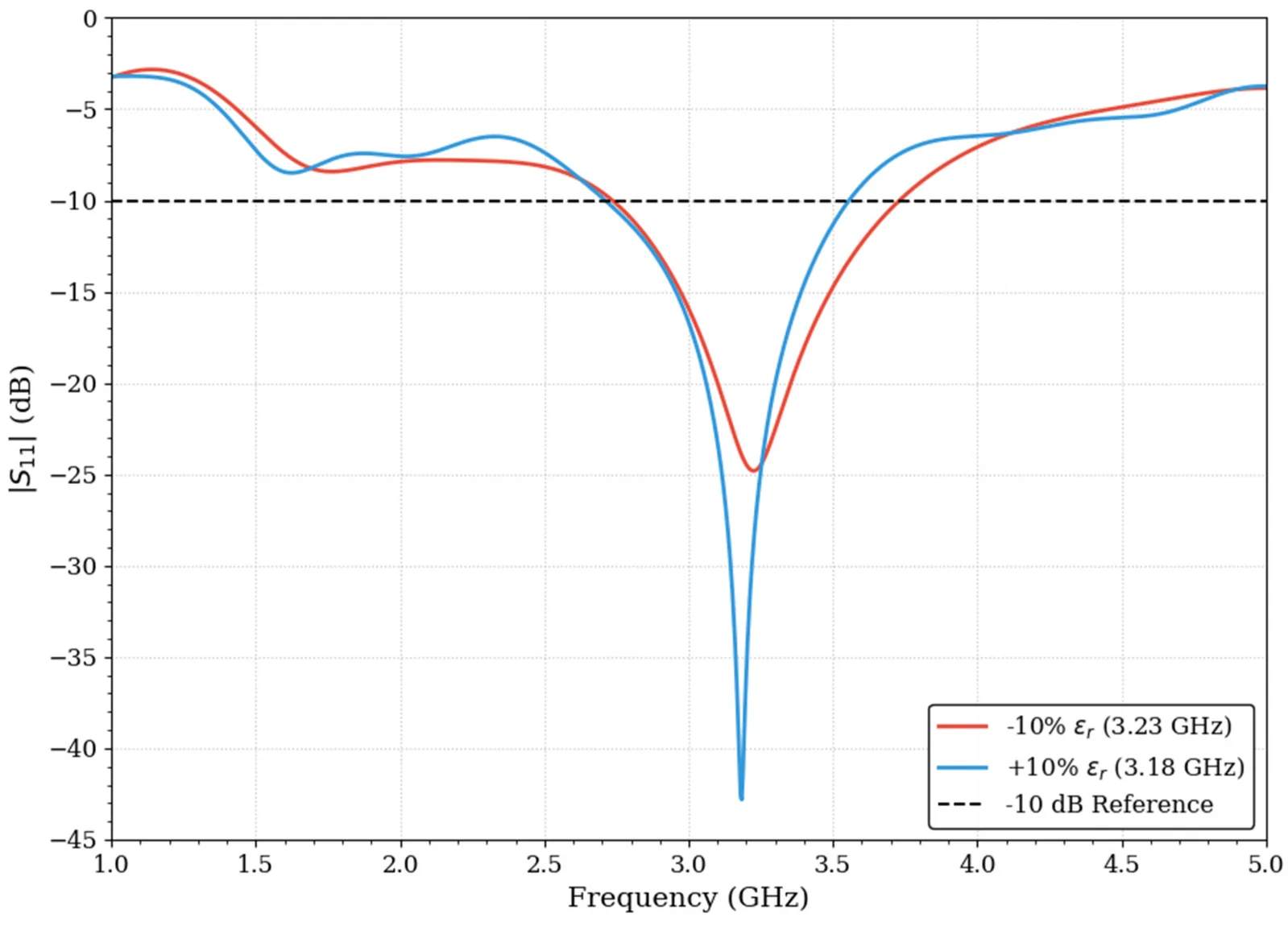
Simulated reflection coefficient ($\left|S_{11}\right|$) demonstrating the antenna’s sensitivity to a ±10% variation in the relative permittivity ($\epsilon_{r}$) of the multi-layer biological tissue phantom. The wideband matching buffer ensures the antenna remains operational across the target sub-6 GHz bands despite the resulting resonance shifts.

**Figure 19:**
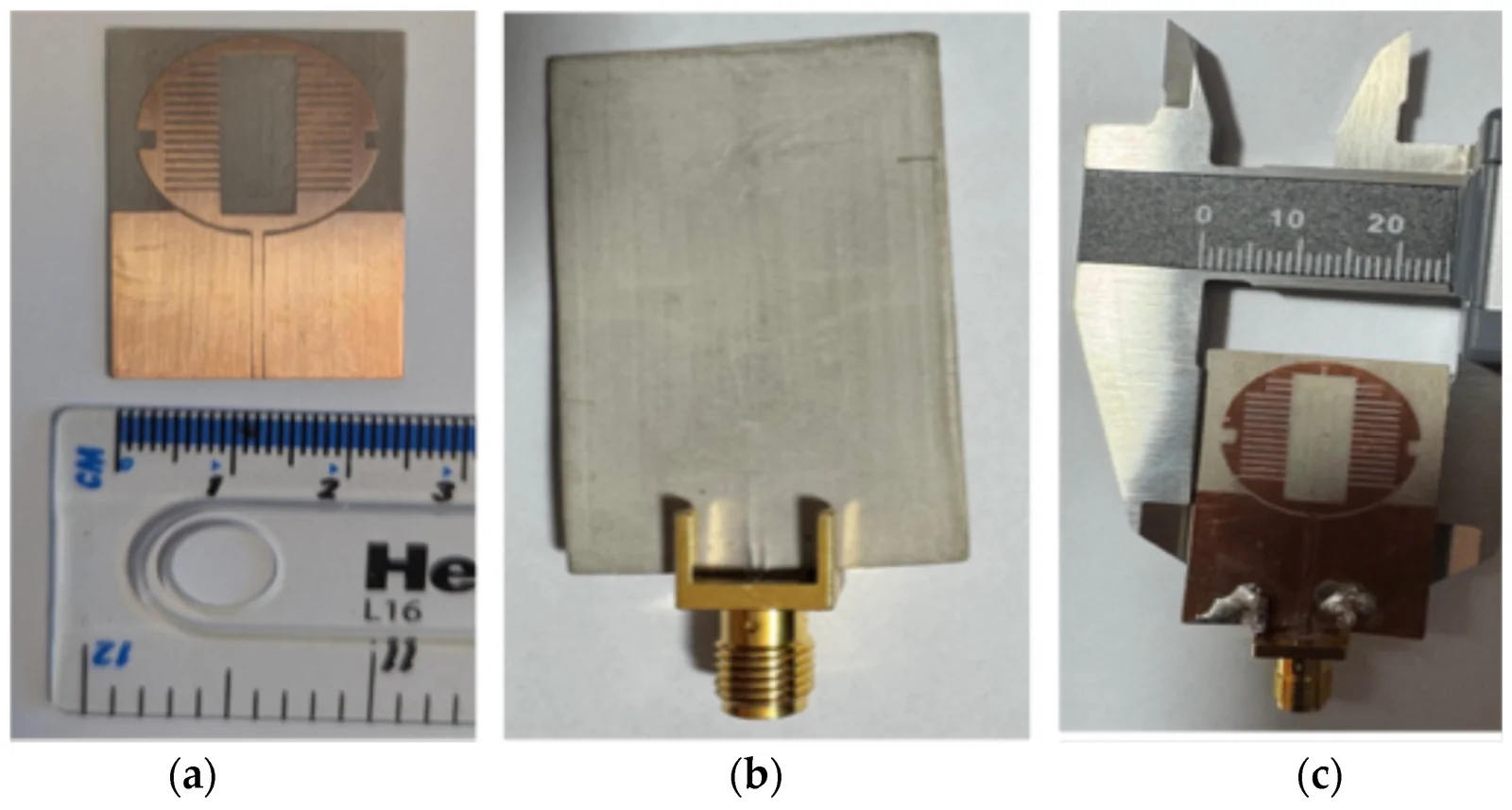
Fabricated wideband circular patch antenna prototype: (**a**) top view showing the interdigital slot structure, microstrip feed line, and front-side partial ground plane, (**b**) bottom view displaying the completely bare, un-metallised Rogers RO3006 substrate and SMA connector mount, and (**c**) dimensional verification using digital callipers (25 mm measured).

**Figure 20:**
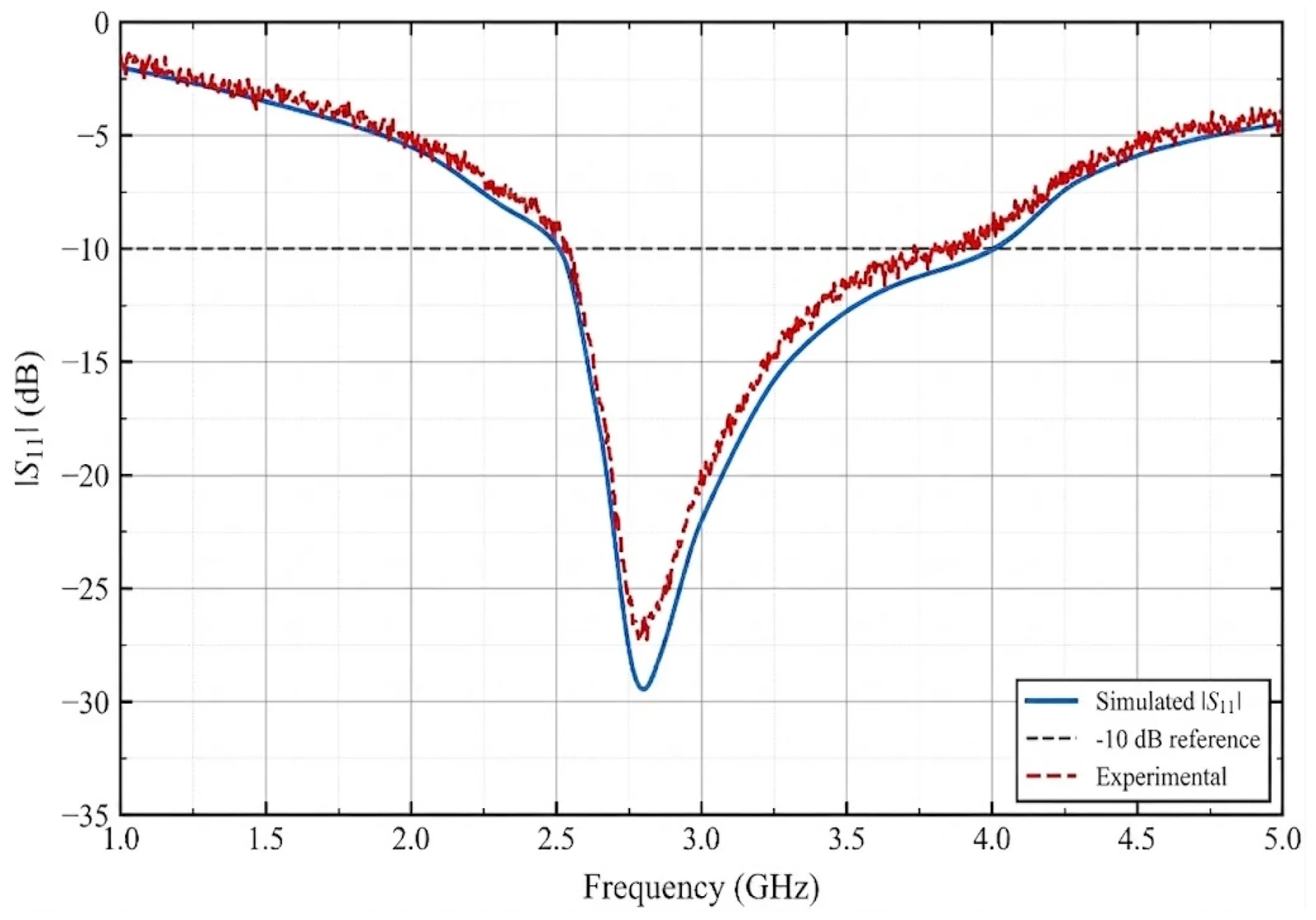
Simulated and measured |S_11_| responses of the proposed antenna (ANT5), demonstrating good agreement between simulation and measurement.

**Table 1:** Design parameters of the proposed antenna (ant5).

Parameter	Value (mm)	Description
*L_sub_*	36.96	Total substrate length
*W_sub_*	25	Total substrate width
*h*	0.8	Substrate thickness
*D*	17.16	Circular patch diameter
*l_f_*	4.68	Interdigital finger length
*w_slot_*	1.17	Slot width
*s*	1.64	Finger pitch
*g*	0.5	Inter-finger gap
*L_g_ × W_g_*	5.69 × 6.65	Partial ground dimensions
*Wf*	0.12	Feed line width
*Lf*	15.97	Feed line length
*W_s_*	2	Feed stub width
*w_finger_*	2.68	Conducting finger width
*tCu*	0.035	Copper thickness

**Table 2:** Comparison of design evolution stages.

Variant	−10 dB BW (GHz)	Fractional BW (%)	Min. |S_11_| (dB)
ANT1	0.28	10	−15.2
ANT2	0.48	17	−18.4
ANT3	0.78	27	−21.7
ANT4	1.08	37	−24.9
ANT5	1.50	46	−29.4

**Table 3:** Circuit model parameters (after optimisation).

Element	Value	Physical Origin
*R_p_*	245 Ω	Radiation resistance
*L_f_*	0.38 nH	Feed inductance
*C_p_*	8.5 pF	Patch capacitance
*L_id_*	1.92 nH	Slot inductance
*C_id_*	0.45 pF	Interdigital capacitance
*L_dps_*	0.88 nH	DPS inductance
*C_dps_*	0.15 pF	DPS parasitic capacitance

**Table 4:** Quantitative comparison of reflection coefficient ($\left|S_{11}\right|$) between full-wave simulation and equivalent circuit model.

Freq. (GHz)	∣S_11_∣ (Full-Wave) [dB]	∣S_11_∣ (Circuit) [dB]	∣Diff∣ [dB]
2.6	−24.97	−24.74	0.23
2.8	−29.40	−28.80	0.60
3.5	−19.43	−18.75	0.68

**Table 5:** Dielectric and Physical Properties of the Multi-Layer Tissue Phantom at 3.38 GHz.

Tissue Layer	Relative Permittivity (ε_r_)	Conductivity (σ) [S/m]	Mass Density (ρ) [kg/m^3^]
Skin	38.0	1.46	1100
Fat	5.28	0.10	910
Muscle	52.0	1.73	1040

**Table 6:** Detuning sensitivity and bandwidth retention of the proposed antenna under varying air gap distances from the tissue phantom.

Air Gap Distance (mm)	Resonant Frequency (GHz)	Frequency Shift (Δ MHz)	−10 dB Bandwidth Maintained (GHz)
2.0	3.42	0	1.03
2.5	3.58	160	0.98
3.0	3.67	250	0.94
4.0	3.79	370	0.88
5.0	3.85	430	0.83

**Table 7:** Detuning sensitivity and bandwidth retention of the proposed antenna under ±10% variation in tissue phantom relative permittivity ($\epsilon_{r}$).

Permittivity Variation	Resonant Frequency (GHz)	Lower −10 dB (GHz)	Upper −10 dB (GHz)	Operational Bandwidth (GHz)
$-10\%\;\epsilon_{r}$	3.23	2.82	3.62	0.80
Baseline (Standard)	3.38	2.82	3.73	0.91
$+10\%\;\epsilon_{r}$	3.18	2.72	3.47	0.75

**Table 8:** Comparison of simulated and experimental antenna results.

Parameter	Simulated	Measured	Deviation (%)
Resonant frequency (GHz)	2.79	2.84	1.8
Return loss (dB)	29.4	26.8	−8.8
Lower −10 dB (GHz)	2.51	2.54	+1.2
Upper −10 dB (GHz)	4.01	3.96	−1.2
Bandwidth (GHz)	1.50	1.42	−5.3
Fractional BW (%)	46.0	43.7	−5.0

**Table 9:** Presents a comparison of the proposed antenna with recently published wideband compact microstrip patch antennas.

Ref.	Substrate (ϵr/h mm)	Size (mm^2^)	Size (λ_0_^2^)	BW (GHz)	FBW (%)	Technique	Gain (dBi)	Gain (Linear)	GBP
[14]	2.2/1.575	60 × 60	0.49 × 0.49	1.45	38.2	Stacked patches	6.8	4.79	182.8
[17]	4.4/1.6	40 × 35	0.35 × 0.31	0.78	27.4	U-slot + DGS	4.1	2.57	70.4
[20]	4.4/1.6	50 × 50	0.50 × 0.50	1.05	32.8	E-shaped patch	5.2	3.31	108.6
[22]	4.4/1	15 × 10.6	0.29 × 0.2	0.58	10.0	DGS	4.4	2.75	27.5
[29]	3.38/0.813	35 × 40	0.33 × 0.38	0.92	30.5	Slot-loaded	4.5	2.82	86.0
[30]	2.2/0.787	45 × 50	0.39 × 0.43	1.32	40.1	Parasitic elements	5.6	3.63	145.6
[36]	6.15/0.64	28 × 32	0.27 × 0.31	0.95	32.1	Interdigital	2.8	1.91	61.2
[44]	4.4/1.6	30 × 30	0.29 × 0.29	0.85	29.3	Fractal slots	3.6	2.29	67.1
[45]	3.55/1.524	42 × 42	0.40 × 0.4	1.25	38.5	Metasurface	5.3	3.39	130.4
This work	6.5/0.8	25 × 37	0.27 × 0.40	1.42	43.7	Interdigital + DPS	3.2	2.09	91.3

## Data Availability

The data supporting the findings of this study are available from the corresponding author upon reasonable request. The datasets contain research materials that are currently being used in ongoing investigations and therefore are not publicly archived at this stage.

## Abbreviations

The following abbreviations are used in this manuscript:

DPS	Defected patch structure
ANT	Antenna
DGS	Defected ground structure
SAR	Specific absorption rate
